# Microbial Community Structure in a Serpentine-Hosted Abiotic Gas Seepage at the Chimaera Ophiolite, Turkey

**DOI:** 10.1128/AEM.03430-16

**Published:** 2017-05-31

**Authors:** Anna Neubeck, Li Sun, Bettina Müller, Magnus Ivarsson, Hakan Hosgörmez, Dogacan Özcan, Curt Broman, Anna Schnürer

**Affiliations:** a Department of Geological Sciences, Stockholm University, Stockholm, Sweden; b Department of Microbiology, Swedish University of Agricultural Sciences, Uppsala, Sweden; c Swedish Museum of Natural History, Department of Paleobiology and Nordic Center for Earth Evolution, Stockholm, Sweden; d Department of Geological Engineering, Istanbul University, Istanbul, Turkey; Goethe University Frankfurt am Main

**Keywords:** Archaea, microbial community structure, bacteria, hydrogen, ophiolite, serpentinization

## Abstract

The surface waters at the ultramafic ophiolitic outcrop in Chimaera, Turkey, are characterized by high pH values and high metal levels due to the percolation of fluids through areas of active serpentinization. We describe the influence of the liquid chemistry, mineralogy, and H_2_ and CH_4_ levels on the bacterial community structure in a semidry, exposed, ultramafic environment. The bacterial and archaeal community structures were monitored using Illumina sequencing targeting the 16S rRNA gene. At all sampling points, four phyla, Proteobacteria, Actinobacteria, Chloroflexi, and Acidobacteria, accounted for the majority of taxa. Members of the Chloroflexi phylum dominated low-diversity sites, whereas Proteobacteria dominated high-diversity sites. Methane, nitrogen, iron, and hydrogen oxidizers were detected as well as archaea and metal-resistant bacteria.

**IMPORTANCE** Our study is a comprehensive microbial investigation of the Chimaera ophiolite. DNA has been extracted from 16 sites in the area and has been studied from microbial and geochemical points of view. We describe a microbial community structure that is dependent on terrestrial, serpentinization-driven abiotic H_2_, which is poorly studied due to the rarity of these environments on Earth.

## INTRODUCTION

Serpentinization is a mineral hydration process that produces H_2_, CH_4_, alkaline fluids, and low-molecular-weight organic compounds and has been suggested to constitute a source of carbon and energy for chemosynthetic life (1–9). Ultramafic, iron-bearing rocks generate H_2_ by splitting water concomitant with iron oxidation. Subsequently, CH_4_ is synthesized via a Fischer-Tropsch-type reaction in which H_2_ reduces CO_2_ to CH_4_ (1–15). Low-temperature abiotic gas seepages on land, such as the Chimaera seep in Çıralı, Antalya Gulf, Turkey, have been identified at only a few sites on Earth (1, 16). The gases issuing from the ophiolitic outcrop are suggestive of formation via low-temperature serpentinization mixed with thermogenic and/or microbial gases. The exhaled gas typically comprises 87% CH_4_, 7.5 to 11% H_2_, 2 to 4.9% N_2_, 0.57% light alkanes, 0.01 to 0.07% CO_2_, and 80 ppm helium (6, 17, 18), and the fluids have pH values between 7 and 12 (19, 20). This gas composition permits both abiotic and biotic CH_4_ production as well as chemosynthetic consumption (21–23). Previous studies of terrestrial serpentinization-driven environments have shown microbial community structures composed of members of the Chloroflexi, Firmicutes, and Proteobacteria phyla, in descending order (1, 3–15). Representatives of the phylum Betaproteobacteria, specifically the Hydrogenophaga genus (which comprises hydrogen-oxidizing, neutrophilic bacteria), are abundant at sites similar to Chimaera, such as the Tablelands ophiolite (1, 10–16), the Cabeço de Vide Aquifer (CVA) (1, 6, 16–18), and The Cedars (6, 17–20).

Chimaera is located in a zone known as the Tekirova ophiolites, which is part of the Antalya Complex; it consists of several north-south trending zones (19–23). This complex and its stratigraphic features, which have been previously described in detail (21–23), show that early Mesozoic rifting, basin formation, passive margin development, and collapse and closure occurred during the Tertiary (Eocene) epoch. In this region, the Beydağları platform is one part of the Mesozoic-Cenozoic carbonate platform and comprises a Paleozoic basement and overlying carbonate sequences. Another carbonate platform, the Kemer Zone, is exposed further east. The basement rock sequence and overlying carbonates of the Kemer Zone are separated by Late Mesozoic ophiolites and mélange units. The western mélange zone is called the Godene Zone and is exposed as tectonic blocks. On the Mediterranean coast, further east, an ophiolite sequence is exposed in the Tekirova Zone (see Fig. S1 in the supplemental material).

The Tekirova ophiolite presents well-preserved ultramafic to mafic cumulate rocks that crop out at Çıralı, where the gas seep is located (24). These rock units are composed of peridotites and gabbro and include serpentinized harzburgites with minor lherzolite, podiform dunites, and chromitites (25). There are three primary source rocks with hydrocarbon generation potential as a part of the sedimentary sequence beneath the Tekirova ophiolite. These areas include Sapandere bituminous shale (Ordovician-Silurian, type I and II organic matter), Beydağları limestone (lower Mesozoic, type II, maturity of 0.4 to 1% reflectance in oil [Ro]), and Pamucakyayla siltstones and coal (Carboniferous, type III, 0.9 to 1% Ro) (21, 26).

Given the difficulties of sampling subsurface crustal environments, an exposed section of an ancient oceanic crust may provide insights into the sources and sinks of energy for microbial communities.

The aim of this study was to investigate the microbial community structures present at the Chimaera ophiolite and to determine the extent to which serpentinization can provide sufficient energy for microbial growth. The microbiology at this site has been somewhat studied before but only with DNA extracted from laboratory enrichment cultures and not with environmental samples (20). This study provides a higher-resolution, detailed study of the community structure at the Chimaera ophiolite using samples directly frozen to −60°C at the site. The study further investigated the influence of the fluid geochemistry of the site on community structure, which has not been done before.

## RESULTS

### Optical microscopy, scanning electron microscopy (SEM), and Raman spectroscopy.

**FIG 1:**
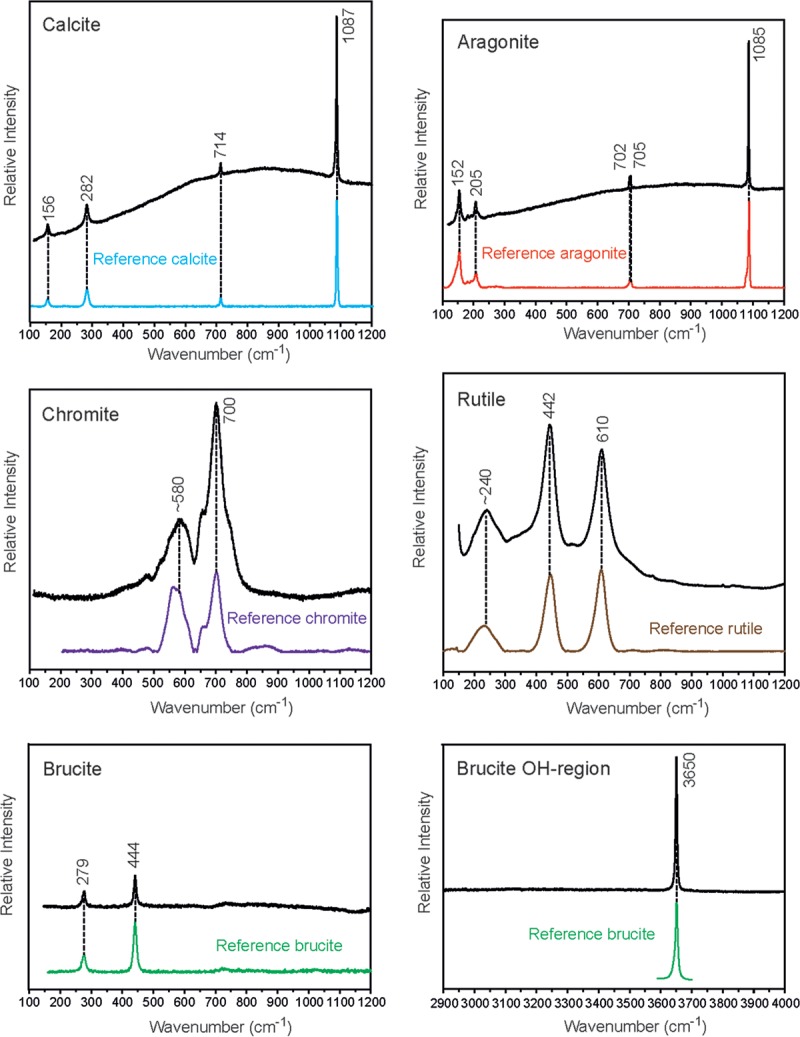
Unprocessed Raman spectra collected for typical minerals in the white and red precipitates from the FG6 and FG7 samples. The minerals were identified by comparison with reference spectra in the 100- to 1,200-cm^−1^ spectral region from the RRUFF project from Downs (59) and in the region above 3,000 cm^−1^ for the OH stretching from Dawson et al. (60).

The sampled sites were similar in mineralogy and consisted of calcite, aragonite, brucite, serpentine, graphitic carbon grains, rutile, iron oxides, and chromite (Fig. 1). Calcite/aragonite and serpentine dominated the mineralogy, but grains of rutile, chromite, iron oxides, and graphitic carbon were scattered within the calcite.

SEM and Raman analyses showed that a white precipitate from site FG6 primarily consisted of calcium carbonates, primarily as aragonite. Site FG7 had a similar aragonite composition but a higher concentration of ferrihydrites, giving the samples a reddish color. Ferrihydrites were also the dominating mineralogy at site IR, whereas carbonates in the form of laminated travertines covered with a thin biofilm dominated site BF. Staining with green fluorescence, used to distinguish between the calcitic and organic material, showed distinct differences between the organic and abiotic layers. Microspheres of 20 to 25 μm in diameter composed of Si, Al, O, and sometimes K were also found. Carbonates could be detected in the surrounding host material but not in the spheres themselves.

### Fluid chemistry.

**FIG 2:**
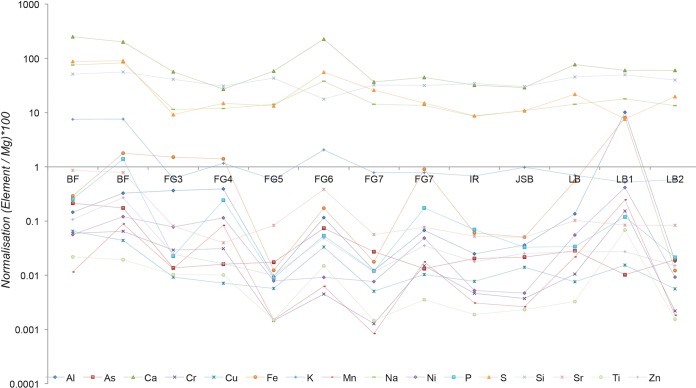
Fluid chemistry overview. All elements were normalized to the amount of Mg and are presented as a percentage of Mg because of the overall dominance of this element at the site. The major elements were close to or above 100%, and the minor elements were close to or below 1%.

The major and minor dissolved elements that were measured using inductively coupled plasma-optical emission spectroscopy (ICP-OES) are presented in Fig. 2 and Table S1 in the supplemental material. The lowest total of dissolved elements was observed in site BF, which had a total elemental concentration (of the measured elements) of 48.1 ± 1.92 ppm. In contrast, the LB1 site had the highest concentration, with a total concentration of 302 ± 12.1 ppm. The fluid chemistry at site FG changed downstream from the individual streams coincident with the change in pH value. The total elemental concentration decreased downstream from 227 ± 9.08 ppm at the top FG3 sample to 143 ± 5.72 ppm at FG6, and there was a slight increase to 190 ± 7.60 ppm at FG7. All FG samples were dominated by Mg (32.4 ± 1.38 to 116 ± 4.64 ppm), except FG6, which was dominated by Ca (73.7 ± 2.95 ppm).

The pH values at site LB increased slowly downstream from the initial value of 7.57 at the source pond to 8.42 at the end of the LB stream. The total elemental concentrations varied from 204 ± 8.16 to 302 ± 12.1 ppm. Mg was the dominant element and varied between 81.9 ± 3.28 and 117 ± 4.68 ppm, followed by Ca (52.2 ± 2.09 to 70.5 ± 2.82 ppm), Si (34.8 ± 1.39 to 58.4 ± 2.34 ppm), S (8.87 ± 0.35 to 17.9 ± 0.72 ppm), and Na (11.7 ± 0.47 to 21.1 ± 0.84 ppm). Among the samples from the area, the highest measured concentrations of Al, B, Co, Cr, Na, Ni, Ti, and V as well as the total elemental concentration were observed at LB1. The IR site had a pH of 7.88, was dominated by Mg (92.5 ± 3.70 ppm), and exhibited high concentrations of Fe (55.8 ± 2.23 ppm), Si (31.9 ± 1.28 ppm), Ca (29.7 ± 1.19 ppm), S (8.09 ± 0.32 ppm), and Na (7.89 ± 0.32 ppm). The BF site had the highest measured pH of 11.35 and the lowest total elemental concentrations. The dominant element at BF was Ca (17.8 ± 0.71 to 23.7 ± 0.95 ppm), followed by Mg (8.85 ± 0.35 to 9.48 ± 0.38 ppm), S (8.00 ± 0.32 to 8.34 ± 0.33 ppm), Na (7.16 ± 0.29 to 7.32 ± 0.29 ppm), Si (4.84 ± 0.19 to 4.97 ± 0.20 ppm), and Fe (∼ 0.03 to 0.16 ± 0.01 ppm). The river passing northeast of the ophiolite (site JSB) had a stable pH of 8.02 and low concentrations of all measured elements, including Mg (77.1 ± 3.08 ppm), Si (23.2 ± 0.93 ppm), Ca (22.3 ± 0.89 ppm), Na (8.37 ± 0.33 ppm), S (8.32 ± 0.33 ppm), and Fe (∼0.04 ppm). The amount of S was below the detection limit at all sites.

### Microbial communities.

The analysis of the bacterial communities generated a total of 1,020,019 sequences after quality trim and chimera check, with a range of 37,341 to 114,792 sequences per sample. The observed number of species (operational taxonomic units [OTUs]) varied between 2,212 and 6,767, with the highest numbers at the BF, JSB, and LB sites (Table 1). Based on the number of species and the Chao1 index, the sequencing covered 47.6 to 65.9% of the total bacterial community.

**TABLE 1:** Summary of observed OTUs, Chao1 values, and Shannon and Simpson indexes at the different sampling points

Sample name	Chao1	No. of OTUs	Shannon index	Simpson index	Total no. of elements	Fe concn (ppm)	pH
BF1	10,698	6,254	11	1	55	711	11
BF2	11,109	6,634	11	1	48	672	11
FG1	9,524	6,030	11	1			
FG2	4,187	2,212	6	1		1,540	8
FG4	4,648	2,712	8	1	220	1,630	9
FG6	8,705	4,973	9	1	132	64	11
FG7.1	6,902	3,697	8	1	188	16	9
FG7.2	9,335	5,169	10	1			9
FG7.3	9,479	5,107	9	1			9
IR	4,842	2,888	7	1	171	56	8
JSB	10,521	6,357	11	1	140	39	8
LB	10,901	6,767	11	1	213	446	8
LB1	10,233	6,577	11	1	302	9,490	8
LB2	11,692	6,722	11	1		11	8
LB2.2	11,913	6,767	11	1			8
SB	7,358	4,851	10	1			

The Shannon and Simpson diversity indices yielded high values, with some variations among the different samples (Table 1). FG (except FG1) and IR sites had relatively low diversity, as illustrated by the lower values of both the Shannon and Simpson indices. The rarefaction curve confirmed the trends, with a higher number of observed OTUs in the BF, JSB, and LB samples (Fig. S3). No correlations were observed between pH and diversity or between the total amounts of trace elements and diversity (correlations of <0.1 for all diversity indices compared to the pH and elemental concentration).

### Phylogenetic analysis.

**FIG 3:**
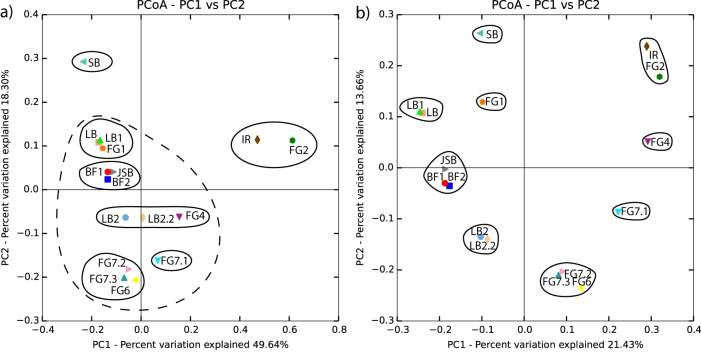
The relative abundance between samples as determined by weighted UniFrac principal-coordinate analysis (PCoA) (a) and the phylogenetic distance determined by unweighted UniFrac PCoA (b). The solid and dashed lines indicate the suggested groupings of the samples.

The phylogenetic composition determined by principal-coordinate analysis (PCoA) of the weighted UniFrac matrices revealed separation among the samples, suggesting phylogenetic distance within the communities at these different sites (Fig. 3a). The plot obtained by unweighted PCoA revealed a similar pattern but with an even clearer separation for some samples (Fig. 3b). Samples obtained from proximal sites, such as LB/LB1, LB2/LB2.2, BF1/BF2, and FG7.2/FG7.3, grouped together, suggesting that the separation between the other samples was due to variations in environmental parameters and not to the sampling approach. Only FG7.1, which was obtained at the same location as FG7.2 and FG.3, deviated from this pattern. Samples FG2 and IR exhibited similar community structures, and JSB grouped with BF in both analyses, whereas SB was separated from the rest of the samples. FG4 and LB grouped together in the weighted analysis but were separated in the unweighted plot, suggesting similar community structures but differences in their abundances.

### Community structure.

**FIG 4:**
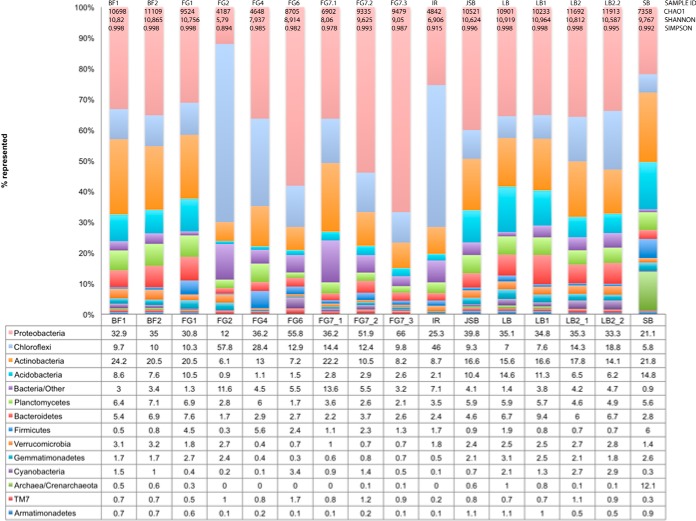
Surface water microbial taxonomic composition at the different sampling sites by phylum.

The abundance of Archaea representatives ranged between 0.10 and 12.1%, with comparably high levels in five (SB, LB, LB1, JSB, and BF2) of the 16 samples, with Crenarchaeota as the dominant phylum (Fig. 4). Phylum comparisons among all samples revealed that approximately 95 to 96% of the bacterial abundance was affiliated with 11 phylotypes (Fig. 3). Members of the Acidobacteria, Proteobacteria, Chloroflexi, and Actinobacteria phyla were predominant in all samples, but the proportions varied among the different samples (Fig. 4). In addition, Bacteriodetes, Firmicutes, Planctomycetes, Cyanobacteria, Deinococcus-Thermus (with the major genus Truepera), and Nitrospirae were present at lower abundances. Proteobacteria was the dominant phylum represented in the majority of the samples, with levels of 21.1 to 66.0% of the total community and with relatively high abundance at the FG6/FG7.2/FG7.3 sites (51.9 to 66.0%). At a few sites, the abundance of Proteobacteria was lower than that of other phyla, including at FG2 (12.0%) and IR (25.3%). At these sites, Chloroflexi was instead the dominant phylum, with a relative abundance of 46.0 to 57.8%. The relative abundance of Chloroflexi members in the remaining samples varied from 5.8 to 28.4%, with the lowest and highest values at sites SB and FG4, respectively. Members of the Actinobacteria phylum were present at levels of 13.0 to 24.2% in all samples except FG1, FG7.2, FG7.3, and IR, which had relative abundances of 6.1 to 10.5%. The relative abundance of Acidobacteria was comparably high at the LB, BF, SB, and FG1 sites (7.6 to 14.8%), whereas this phylum was represented at small percentages (0.9 to 2.1%) at the remaining sites. In addition, an unidentified phylum was identified at levels of 0.9 to 13.6%, with the highest abundance in FG1 and FG2.

Proteobacteria were generally dominated by the Alphaproteobacteria class (6.4 to 25.2%, where SB had the lowest relative abundance and FG4 had the highest) and Betaproteobacteria (0.7 to 26.7%, where FG2 had the lowest relative abundance and FG7.3 had the highest), but members of a few orders of the sulfate-reducing bacteria Desulfovibrionales were found at FG4 and FG1. Deltaproteobacteria were found in relative amounts of 0.8 to 4.9% (where FG6 had the lowest relative abundance and SB had the highest), followed by Gammaproteobacteria at 1.2 to 19.1% (where FG2 had the lowest relative abundance and FG6 had the highest) (Fig. S4). The primary orders (Table S3) represented within Deltaproteobacteria and Gammaproteobacteria were Rhizobiales (2.8 to 11.9%), Burkholderiales (0.5 to 23.5%), Myxococcales (0.5 to 5.3%), and Methylococcales (0.1 to 7.6%). In addition, members of the orders Rhodobacterales (0.6 to 7.4%), Rhodospirillales (0.8 to 4.4%), and Sphingomonadales (0.6 to 10.2%) within Alphaproteobacteria were present at all sites. The Deltaproteobacteria consisted primarily of members from the Myxococcales order (0.5 to 5.3%), and the Xanthomonadales order (0.1 to 14.6%) was present at high abundance within the Gammaproteobacteria class and was particularly abundant in FG6 (14.6%). The members of the genus Hydrogenophaga within the phylum Betaproteobacteria were found at all sites except FG1, FG2, FG4, and SB, with the highest level at FG7.3 (0.1% to 14.1%).

The Actinobacteria phylum was typically dominated by the Actinomycetales order (2.2 to 12.3%) but was also represented by Gaiellales (0.1 to 7.9%), Rubrobacterales (0.1 to 4.5%; Rubrobacterales was absent at FG2, FG4, and IR), and Solirubrobacterales (0.5 to 6.9%). Acidobacteria were represented by members from the orders RB41 (0.2 to 6.3%) and iii1-55 (0.1 to 5.3%).

The composition of the Chloroflexi phylum varied greatly among the different sites. Among members of the Chloroflexi phylum, the order SBR1031 (0.6 to 16.1%) in the class Anaerolineae often dominated. However, the members of the order Chloroflexales (0.2 to 1.5%; absent in IR, FG2, and SB) within the class Chloroflexi and JG30-KF-CM45 (0.7 to 2.7%) within the Thermomicrobia class were also observed. In addition, the orders Thermogemmatisporales (0.1 to 24.1%; absent at LB, BF1, BF2, LB1 and SB) and the class Ellin6529 (0.1 to 2.8%) were observed. At sites FG2 and IR, which had very high relative abundances of Chloroflexi, the orders Thermogemmatisporales (13.3 to 24.1%) and unclassified Ktedonobacteria (22.1 to 26.4%) were dominant. The primary Archaea phylum identified was Crenarchaeota, which represented less than 1% in all samples except for SB, wherein this phylum represented as much as 12.1%. In all cases, independent of abundance, Nitrososphaera was the only represented genus. At FG7, the Archaea phylum Euryarchaeota was detected, in addition to Crenarchaeota, representing 0.2% of the total community, and Methanobacterium was the only represented genus at this site. At sites FG2, FG4, and IR, no representatives of the Archaea were detected.

The phylum Acidobacteria was dominated by members of the order Actinomycetales (2.2 to 12.3%), followed by RB41 (0.2 to 6.3%), Solirubrobacterales (0.5 to 6.9%), iii1-15 (0.1 to 5.3%), and Solibacterales (0.2 to 1.4%). Bacteria capable of aerobic methane oxidation (MO), such as representatives of the taxa Beijerinckiaceae, Balneimonas (Alphaproteobacteria), Methylibium, Methyloversatilis (Betaproteobacteria), and Methylococcaceae (Gammaproteobacteria), were detected in all samples (0.9 to 5.5%), and their abundance was highest at FG4, where MO bacteria were found at a total level of >8%. Sulfate reducers from the family Thermodesulfovibrionaceae were found at FG6, and the order Desulfovibrionales was found at LB, JSB, FG1 and FG4.

### CCA.

**FIG 5:**
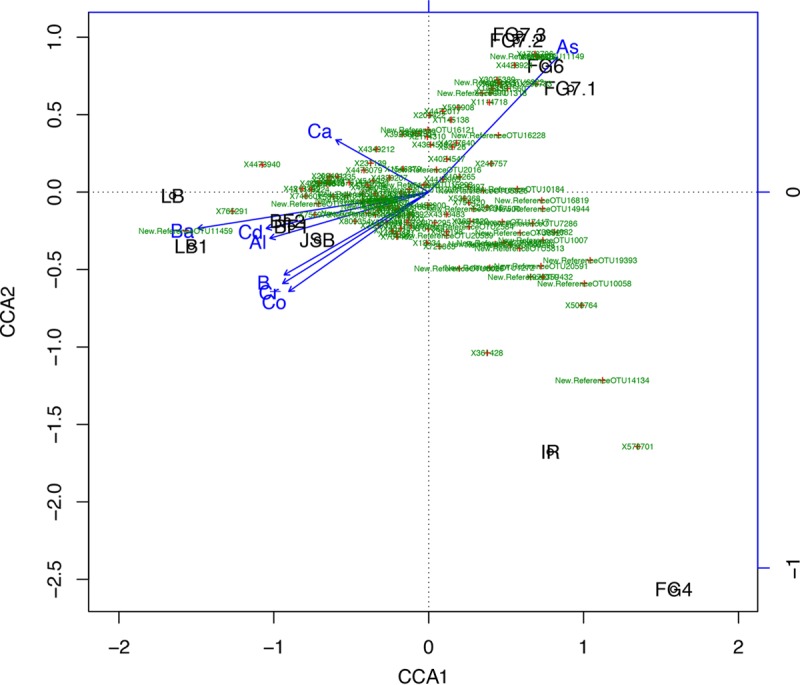
Canonical correspondence analysis (CCA) at the OTU abundance level of >0.1% using all elemental data and pH. In the plot, the black labeling indicates the sampling site, green numbers are OTU numbers (with red dots in the center), and blue arrows represent the metal data.

Canonical correspondence analysis (CCA) was performed to identify correlations between the microbial community and different chemical parameters. The OTUs corresponding to >0.1% relative abundance and 26 metals were included in the CCA. Three major clusters were obtained, and correlations were observed between the FG6/FG7 cluster and As and between the LB/JSB/BF clusters and Ba, Cd, Al, B, and Cr (Fig. 5).

The systematic removal of variables revealed new correlations such as the correlation of FG6/FG7 with Cu (Table 2 and Fig. S5 and S6). When only seven variables were used in the CCA plot, a weak correlation between pH and FG6/FG7 was observed (Table 2 and Fig. S5 and S6). The metals that significantly correlated with the bacterial community composition (Fig. 5, longer arrows) when all 26 measured elements were included were As, Ba, Cd, Al, B, Cr, and Co.

**TABLE 2:** Correlations between the bacterial community structure and environmental variables

No. of variables	Element(s) present at:		
	FG6/FG7	IR/FG4	LB/JSB/BF
26	As		Ba, Cd, Al, B, Cr, Co
25	Cu		
24			Fe
23		K	
22		Li	
19		Mg, Mo	Mn
18	Na		
14	S	P, Pb	Ni
10			V, Ti, Sr
7^*a*^			Zn

a A correlation between pH and FG6/FG7 was observed when only seven variables were used.

## DISCUSSION

This study presents 16S rRNA sequence data from as many as 16 different sites at the Chimaera ophiolite and represents an expansion from an earlier study investigating six sampling sites (20). In contrast to the previous work, this study, in addition to a description of the community structures at the surface of the ophiolite, also presents data from surrounding rivers. Moreover, DNA was extracted directly from samples taken from the site and not, as in the previous study, from selective enrichment cultures. Thus, this study will show a more representative map of the community structure at the Chimaera ophiolite. The difference in sampling handling may explain differences in the results between the two studies. In contrast to the study of Meyer-Dombard et al., we identified, for example, representatives from the Archaea and many potential anaerobic bacteria.

Comparison with similar studies of other serpentinization-driven, alkaline environments, such as The Cedars (in coastal northern California) (6), the Cabeço de Vide Aquifer (CVA) (Portugal) (1), and the Tablelands (western Newfoundland, Canada) (10), also shows differences in community compositions. At CVA, Tablelands, and The Cedars, the main sequences were attributed to H_2_-producing Clostridia (phylum Firmicutes) and H_2_-consuming Hydrogenophaga (phylum Betaproteobacteria), whereas in Chimaera, the major sequences were attributed to Alphaproteobacteria, Chloroflexi, and Actinobacteria. Both Hydrogenophaga and members of the class Clostridia were found at Chimaera.

Alkaline springs such as those at Chimaera, Oman, and Voltri Massif are commonly characterized by high pH, Ca, H_2_, and CH_4_ and a depletion in transition metal and Si contents (20, 27, 28). Based on the dissolved-element chemistry, our results are consistent with previous alkaline spring data (6, 29, 30) indicating that the cooccurrence of high pH values and Ca ions indicates an active serpentinization area, whereas the cooccurrence of Fe, Cr, Mn, Co, and Ni with Mg suggests chemical weathering. In addition, cooccurrence of Mg with Ba, Al, and Ti was observed in this study, reflecting ongoing weathering of the ophiolite. The pH values of the FG and LB streams differ because the FG stream has an oscillating pH downstream, whereas the LB stream exhibits a gradual increase in pH. Our data indicate a general increase in downstream pH, in contrast to the downstream decrease in pH observed by Meyer-Dombard et al. (20) and others (27, 28). This discrepancy may be attributable to differences between the measured streams but also a mixing of meteoric, groundwater, and serpentinization fluids. The streams on the ophiolite are only occasionally present during the rainy season and dry out completely in the interim. Although the processes should be similar, many factors may have influenced the changes in pH, such as more vigorous serpentinization of higher water flow. The higher concentrations of metals downstream of the FG stream coincide with the lower pH due to higher weathering rates and the generally higher solubility of metals at lower pH. At the FG stream, high concentrations of Ca ions coincide with high pH, which is considered indicative of active serpentinization (20, 31–33). Active serpentinization in low-temperature environments usually generates substantially reducing conditions due to low Si activity and, in this case, a high Ca^2+^ concentration. The area is supersaturated with Ca^2+^ with respect to calcite, which was found in the majority of the rock samples. The source of the LB stream was situated at the top of the ophiolite and was likely mostly meteoric water, which explains the low pH of this site. The stream meanders downhill, reacts with the ultramafic rocks, and becomes increasingly alkaline. The upper parts of the FG stream have similar features, but downstream at the FG5 site, a second source of water emanates from the channel of a doused flame, explaining the dramatic increase in the pH to 11.03. Downstream of FG5, there is a decrease in pH similar to that observed in a study by Meyer-Dombard et al. (20) and is likely caused by carbonate and ferrihydrite precipitation and a concomitant pH decrease. CCA suggested correlations between the phylogenetic distributions at FG6 and FG7 (FG7.1, FG7.2, and FG7.3) and alkaline pH (see Fig. S6 in the supplemental material).

Members of the Hydrogenophaga genus were observed in FG6/FG7, in contrast to results with the other samples. This genus has been described as a Gram-negative, aerobic, chemoorganotrophic or chemolithoautotrophic H_2_-consuming bacterium that can partly perform heterotrophic denitrification. Hydrogenophaga grows at temperatures of approximately 30 to 40°C and at pH values of 6.5 to 9.0 (20, 34). The FG samples were all situated in the middle of the ophiolite, far from any vegetation and close to H_2_ and ever-burning flames. The increased presence of Hydrogenophaga at the FG6/FG7 sites and the apparent correlation with pH may have many explanations. The high relative abundance of Hydrogenophaga in FG6/FG7 compared with that of the other sites and the slight correlation between pH and FG6/FG7 (Fig. S5) revealed by CCA do not necessarily indicate that Hydrogenophaga is more strongly correlated with pH but, instead, that the community structure as a whole is somewhat correlated with pH. Hydrogenophaga can grow at pH values of up to 9.0 (35), but one study of alkaline shallow groundwaters in Lake Calumet, Chicago, IL (36), did show that Hydrogenophaga was active at pH 11.8, consistent with the presence of this genus in FG6, which has a pH of 10.7.

The Si composition of the area fluctuated between ∼5 and ∼42 mg/liter, compared with the global average composition of stream water and seawater of 13.9 and 5.99 mg/liter, respectively (37). The Si at the Chimaera site is undersaturated with respect to amorphous Si, and no opaline phases were detected by Raman spectroscopy or environmental SEM (ESEM). This finding is consistent with the presence of the secondary mineral brucite, which indicates low Si activity in the serpentinization fluids and, consequently, brucite precipitation (7). There is a negative correlation between the diversity index and concentration of Si, Fe, and other metals among the FG samples. It can also be noted that at FG6, where there is a general drop in elemental concentration, the pH rises to almost 11, and the abundance of Methylocaldum (Gammaproteobacteria) increases dramatically. This increase in Methylocaldum, which is a thermophilic, methanotrophic bacterium, is likely due to the proximity to the doused flame, where there is a continuous seepage of CH_4_. The negative correlation between diversity and Si concentration is thus likely an indirect correlation that is coupled to water mixing and gas seepage rather than an actual correlation between elemental concentration and diversity.

At site BF, where microstromatolites with layers of calcite alternated with organic material were found, the diversity index is high. Diversity is high at the BF microbial mat, with an increased abundance of representatives of the Acidobacteria, Cyanobacteria, and Crenarchaeota, common phyla in terrestrial, hot spring microbial mats with a low-salinity and low-sulfate composition (2).

### Diversity of the bacterial communities and comparison to geochemistry.

The highest diversity index was observed at the calcite-dominated BF1 and BF2 sites, whereas the lowest diversity index was observed at FG2 (serpentine) and IR (ferrihydrites) (Table 1). Within the low-diversity sites, the members of the Chloroflexi phylum were present at a relatively high abundance, whereas Proteobacteria dominated at the high-diversity sites. Chloroflexi have been suggested to be able to adapt to changing redox conditions due to a versatile metabolism that enables growth under anaerobic as well as aerobic conditions (38) and competition for labile carbon species. Coupling between Chloroflexi and low diversity was also documented at The Cedars, an ultramafic serpentinized peridotite (6). In a microbial diversity study of a Venezuelan orthoquartzite cave, the presence of Chloroflexi members was associated with nutrient limitation (39), as confirmed in other studies (38, 40) in which representatives from this phylum (particularly Ktedonobacterales) were shown to be well adapted to growth under nutrient limitation. Adaptation to changing redox conditions among members of the Chloroflexi phylum may also affect their advantages in specific systems such as the Chimaera ophiolite (38). All BF samples had very high P concentrations (24.2 to 123.3 ppm), which may explain the higher microbial diversity at this sample site. Similar P levels were measured at other sites, but since the amount of Fe was extremely high at the IR site, the amount of available P was likely limited due to the strong P adsorption onto Fe oxides (41). The Fe/P ratio (in parts per million) was 1,110 at the IR site, whereas all the other sites had Fe/P ratios of 1.14 to 53.3. This finding suggests strong limitation in P at site IR, which might confer a competitive advantage to Chloroflexi members (39). No other particular chemical properties were identified at the BF sites that could explain the high diversity. There were, however, small terracettes filled with standing water, which may have facilitated the buildup of biofilm and thus protected against desiccation and chemical gradients, which in turn possibly could promote a higher diversity.

The population compositions of Chloroflexi differed among the samples: FG samples had a higher relative abundances of representatives of thermophilic orders such as the Anaerolineales, Caldilineales, and SHA-3, whereas the lower-temperature sites were dominated by N_2_-fixing members of the Chloroflexi phylum, such as Ellin6529 and filamentous anoxygenic phototrophic Chloroflexales. This Chloroflexi distribution is consistent with the compositional allocations of the different sampling sites because FG is situated at or near burning flames. Suzuki et al. (6) reported that the low species richness at The Cedars was associated not only with Chloroflexi but also with Firmicutes. In contrast, at Chimaera, the abundance of Firmicutes was highest at SB, FG4, and FG1, where the diversity was high.

The abundance of representatives of the phylum Actinobacteria was considerably lower at FG2, FG6, FG7.2, FG7.3, and IR, where the layer of soil was very thin or nonexistent. Because members of the order Actinomycetales (the dominant order of Actinobacteria at Chimaera) are usually found in soil and degrade cellulose and other plant polysaccharides (42), Actinobacteria levels are expected to be lower at sites with little influence from plant and soil material. Similarly, Acidobacteria members are well represented in soil (43) and have lower abundance at sites with little or no soil, such as FG2, FG4, FG6, FG7.1, FG7.2, FG7.3, and IR.

Hydrogen-oxidizing Hydrogenophaga bacteria were found at all sites except FG1, FG2, FG4, and SB, and these organisms have been previously found in similar environments, such as the Tablelands serpentine-hosted alkaline seep (10), at The Cedars serpentinizing environment (6), and at the serpentinization-driven subterrestrial alkaline aquifer Cabeço de Vide Aquifer (CVA) (1). It is not clear if these H_2_-oxidizing bacteria use abiotically produced H_2_ or if H_2_ is produced through fermentative or photosynthesizing activity. However, all sampled sites had extremely low relative abundances of typical H_2_-producing bacteria, such as Cyanobacteria, members of the Clostridia class or the Enterobacteriales, Bacillales, Rhodobacterales, or Thermotogales order, suggesting an abiotic source of H_2_. Previous measurements at Chimaera have indicated that at least CH_4_ has a primarily abiotic origin (16, 18), and because of the low abundance of potential H_2_-producing bacteria as well as the active serpentinization at the site, it is likely that H_2_ has an abiotic source as well. Alkaliphilic bacteria such as those of the genus Silanimonas or Alkaliphilus from the class Gammaproteobacteria or Clostridia, respectively, were found in both CVA and The Cedars (6) and were not found at Chimaera. No known extreme alkaliphiles were found at Chimaera, but bacteria such as those of the Rhizobium genus (class Alphaproteobacteria), which can grow under moderately alkaline conditions, were found at some of the sampled sites.

Nitrogen-fixing bacteria, such as members of the Rhizobiales and Rhodobacterales orders (class Alphaproteobacteria) and the Cyanobacteria phylum, were found in large amounts in many of the samples. Meyer-Dombard et al. (20) did detect Thermobacillus and Anoxybacillus in their enrichment cultures from Chimaera, but these groups were not observed in our study. Nitrogen-fixing and denitrifying bacteria were not measured or detected at The Cedars (6) but were found at CVA, with members of primarily the Nitrospira group (phylum Nitrospirae) (1) represented. The isotopic composition of N_2_ at Chimaera was measured by Meyer-Dombard et al. (20) and was approximately 3‰, suggesting low activity from nitrogen-fixing metabolism in the area. The authors proposed that the nitrogenase genes (*narG* and *nirS*) detected by PCR at the site might have been inactive at the time of sampling, resulting in a high positive isotopic value of N_2_. However, positive nitrogen isotope values were detected in all measured solids, as expected in an active nitrogen cycle ecosystem. In our study, a high abundance of nitrogen-fixing bacteria was detected, which may explain the positive nitrogen isotopic values measured by Meyer-Dombard et al. (20), and this result is consistent with their detection of nitrogenase genes. The abundance of aerobic methane oxidizers was highest at FG4, which is reasonable because FG4 is situated near a source of gases and fluids.

The two larger river sites (JSB and SB) have very different community structures: JSB more closely resembles the LB samples (similar compositions of the typical soil community profiles [44]), whereas SB had a much higher relative abundance of the archaea Nitrososphaeraceae, which are found in low-flux geothermal soils (45) and in the serpentine-hosted hyperalkaline springs of Voltri Massif (46) but not in the Tablelands or The Cedars serpentine environments (1, 6). Nitrososphaeraceae are ammonium-oxidizing, thermophilic archaea, and their presence in SB and not in JSB is likely due to the proximity of the SB river to the ophiolite. The SB river flows very close to the ophiolite, whereas the JSB river is more distant, which would likely result in a higher abundance of geothermally influenced community structures.

### CCA and UniFrac matrices.

Weighted and unweighted UniFrac distance matrixes are commonly used for microbial community structure analyses (47). The unweighted distance analysis considers only information on the presence or absence of species and counts the fraction of phylogenetic branch length for each community, whereas the weighted distance analysis uses species abundance information and weighs the branch length with abundance difference. However, these two measurements may assign too much importance to rare lineages, which may distort the phylogenetic and relative abundance measurements. Canonical correspondence analysis (CCA) was therefore also used for phylogenetic comparisons between the sampling sites. The difference between the weighted and unweighted UniFrac matrices suggests similar OTUs but different abundances within the samples. A comparably clear separation between the samples was obtained with the unweighted analysis. The results of the UniFrac analysis are similar to those shown in Fig. 4, showing only differences in relative abundances of different phyla. One of the major differences between the different analyses is the clustering of IR/FG2 and IR/FG4 in UniFrac and CCA, respectively. Using UniFrac weighted analyses gives a similarity between IR and FG2, a result not obtained with CCA, where a separation is seen. This difference in result is likely due to the geochemical influence in CCA, which is not considered in UniFrac. The differences in correlations between phylogeny and biogeochemistry observed for the different sites in the CCA plot can be divided into three groups. The sites closer to the edges of the ophiolite (JSB, LB, and BF) have a much higher diversity and a stronger coupling to variations in the trace metal geochemistry. The sites in the middle of the ophiolite have a stronger correlation to some rare elements such as As and Cu, elements that might influence if the overall community structure. The FG4 and IR cluster is most likely the result of phylogenetic similarities rather than the influence of geochemical parameters on the different groups.

In the FG6/FG7 cluster, the bacterial family that was most strongly coupled with the geochemical metadata was Comamonadaceae (class Betaproteobacteria), which exhibit resistance to As (48). Copper was associated with the presence of Truepera, a genus within the family Trueperaceae from the phylum Deinococcus-Thermus, documented to be resistant to environmental hazards (49). This genus was present in the samples FG6, FG7, LB, BF, JSB, and FG6, all of which had relatively high concentrations of Cu. The IR/FG4 cluster was associated with the order SBR1031 and the family SHA-31 within the Anaerolineae class, members of which have metabolic ability to produce H_2_ (50). Other bacteria associated with the IR/FG4 cluster were Rhodococcus ruber (an anaerobic, hydrocarbon-degrading bacterium) (51), Phycisphaeraceae (anaerobic, nitrate-reducing marine bacteria (52), and Methylocaldum (a thermophilic methanotroph) (53, 54). The abundance of known anaerobic bacteria was higher at IR/FG4 than at the other sites, which were dominated by aerobic bacteria, possibly due to the anaerobic microenvironments at the sites or higher release of H_2_. The LB/JSB/BF cluster was primarily associated with Nitrososphaera, Crenothrix (which have MO and iron-oxidizing capabilities [55, 56]) and some members of the genus Geodermatophilus (44, 57). These sites (LB/JSB/BF) had a higher relative abundance of typical soil bacteria (44) than the other sites. This result is likely a consequence of the vicinity to the borders of the ophiolitic outcrop, which may influence the community structures through geochemical as well as microbial input from outside the outcrop. The sulfate-reducing bacterium in the Thermodesulfovibrionaceae family found at FG6 is a thermophilic, often obligate anaerobe that has been observed in alfalfa-rice soils (58). The presence of Thermodesulfovibrionaceae at FG6 may be explained by the proximity to the ever-burning flames but not by the S content since all ICP analyses indicated that the S content was below the detection limit. The presence of other sulfate-reducing bacteria such as members of the order Desulfovibrionales at LB, JSB, FG1, and FG4 suggests that S is present at the sites although our data did not indicate the presence of S. However, Meyer-Dombard et al. (20) measured major ions and determined that SO_4_^2−^ was present at concentrations of approximately 29.1 ppm, which supports our results indicating the presence of sulfate reducers.

### Conclusions.

The microbial community structure at the Chimaera ophiolite is similar to that of other terrestrial, serpentine-driven ecosystems and features H_2_ oxidizers, methane oxidizers, and thermophilic bacteria. Nutrient limitation and low diversity were associated with Chloroflexi members, as observed in similar environments. Some metal-resistant and radiation-resistant bacteria were detected at the site, consistent with the geochemical and environmental properties of the samples. Coupling between the special geochemistry of the site (methane and hydrogen seeps and ever-burning flames) and bacterial community structures was observed, such as the presence of methane oxidizers, hydrogen oxidizers, trace metal-resistant bacteria, and thermophilic bacteria as well as the presence of archaea. A clear phylogenetic difference was observed between the different sites, where samples from the middle of the ophiolite, with strong pH gradients and absence of soil, showed a lower diversity as well as differences in community structures. Samples closer to the edges of the ophiolite had a stronger soil-like microbial distribution and higher diversity. Some samples showed a very different microbial composition where Chloroflexi members dominated the bacterial community. Archaea were present in low relative abundance in all samples except one from the larger rivers passing by the outcrop on the northern side, where almost 12% of the relative abundance was represented by archaea.

Future studies include functional analyses in order to more strongly couple the geochemical data to the microbial data, especially when it comes to the coupling between CH_4_, H_2_, and populations, including the use of specific primers to investigate archaea in more detail.

## MATERIALS AND METHODS

### Samples.

A total of 16 samples were obtained at different sites, as follows (see Fig. S2 in the supplemental material). The JSB site was a river passing north of the exposed ophiolite (pH 8.02). The SB site was a river passing through the carbonates and close to the southern part of Chimaera (pH 9.47). The LB site was a small stream that meanders over the ophiolite that has an upwelling source uphill. Four samples were collected at the source of this stream: LB, LB1, LB2, and LB2.2. LB and LB1 were collected at approximately the same sites with a pH of 7.57, and LB2 and LB2.2 were collected at approximately the same sites with a pH of 8.00. The FG site was a small stream that meanders over the ophiolite and has an upwelling source uphill and another source at the exposed sampling site (Fig. S2a to c). Eight different samples were collected from this stream: FG1 at the fluid source; downhill at FG2, FG4 (which is fed by another fluid source), and FG6; and three samples from FG7 (FG7_1, FG7_2, and FG7_3) collected at almost the same spot. The pH values were 7.49 at FG1, 7.43 at FG2, 9 at FG4, 10.70 at FG6, and 8.66 at FG7. The BF site was a small site with travertines and biofilms at the end of a small stream (Fig. S2d). Two samples were collected in almost the same spot, BF1 and BF2, with a pH of 11.4 The IR site was a small stream that meanders over the ophiolite and has an upwelling source uphill and sediment with a strong rusty color (pH of 7.9).

### Sampling area.

**FIG 6:**
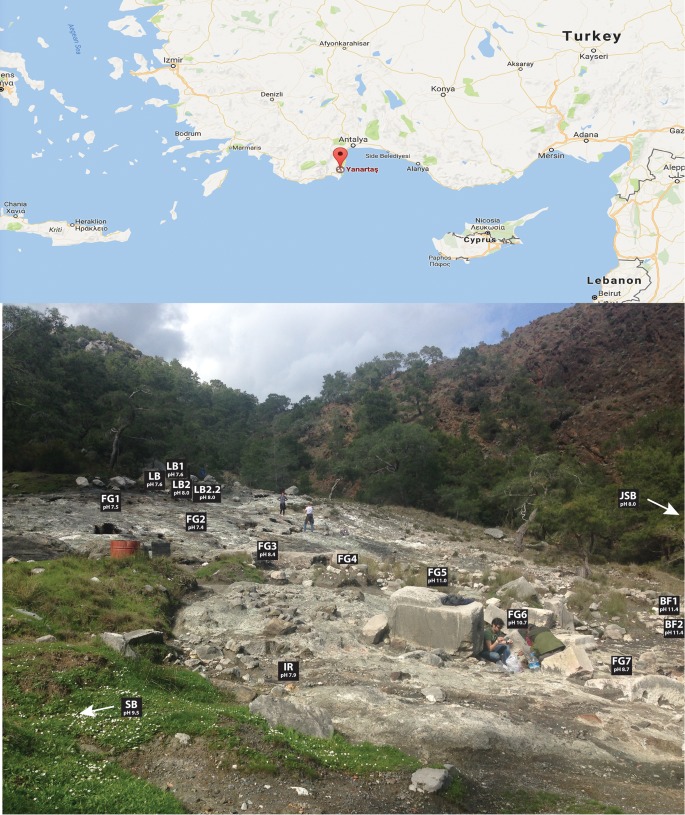
Location of the Chimaera ophiolite. The upper panel shows the location on the map (map of Yanartaş, Antalya, Turkey [Google Maps], accessed 6 March 2017), and the lower panel shows the sampling sites on the ophiolite. The samples SB and JSB are located outside the picture.

The sampling sites are situated at an exposed area located on the Tekirova ophiolite outcrop, which is north of Çıralı, Antalya, Turkey (Fig. 6). Flames of burning gas are scattered around the site, and three small fluid seeps meander their way downward over the outcrop. The site is mostly dry during the year, but during the rainy season (November to February), small streams and ponds of standing water are present not only on the primary sampling site but also close to other sampling sites located at higher elevations. On-site pH measurements were obtained using a hand-held pH meter (pHTestr 30; Thermo Scientific, Eutech Instruments Pte, Ltd., Germany), with an uncertainty of approximately 0.2 pH units.

### Sample collection.

Fluid samples for trace element analyses were collected from BF1, BF2, FG2, FG4, FG6, FG7.1, IR, LB, LB1, and LB2 by withdrawing fluids using sterile syringes. The samples were then injected into acid-washed 10-ml plastic tubes (pretreated with 5 μl of concentrated HNO_3_) and sealed carefully. DNA extraction samples were collected from all sites using sterile spoons, needles, and syringes. Sediment samples (LB, LB2, FG1, FG2, FG4, FG6, FG7_3, BF2, and IR) were scooped into sterile plastic tubes and immediately placed in a container filled with dry ice for freezing at −60 to −80°C. Some samples (JSB, SB, FG7.1, FG7.2, BF1, LB1, and LB2.2) were filtered through a sterile, vacuum-driven (with a hand-held pump) filtration system (Stericup; Merck Millipore, Germany). The filter was removed, placed in a sterile plastic vial, and frozen on dry ice. Solid samples for mineral analyses were collected using a rock hammer and plastic bags. The sediments collected for mineral identification were scooped into sterile plastic tubes with an acid-washed plastic spoon.

### Optical microscopy, ESEM, and Raman spectroscopy.

Thin sections were prepared (VanPetro Petrographics, Ltd., Vancouver, Canada) from the FG6, FG7, BF, and IR sites. All thin-section samples were optically (under regular and fluorescence light) examined using a Leitz DMRBE fluorescence microscope. The surface composition and topography were analyzed semiquantitatively on an XL30 environmental scanning electron microscope (ESEM) with a field emission gun (FEG). The ESEM was equipped with an Oxford x-act energy dispersive spectrometer (EDS), a backscatter electron detector (BSE), and a secondary electron detector (SE). Peak and elemental analyses were performed using INCA (isotopomer network compartmental analysis) Suite, version 4.11, software. None of the samples were coated.

Mineralogical analyses were performed using a Horiba Scientific LabRAM HR 800 laser Raman confocal spectrometer equipped with a multichannel air-cooled charge-coupled-device (CCD) detector. An Ar ion laser (λ = 514 nm) was used as the excitation source. The instrument was integrated with an Olympus microscope, and the laser beam was focused to a spot of 1 μm with a 100× objective and a spectral resolution of approximately 0.3 cm^−1^. The instrument was calibrated using a neon lamp and the Raman line (520.7 cm^−1^) of a silicon wafer. Instrument control and data acquisition were performed with LabSpec, version 5, software and known databases such as RRUFF (59, 60).

### Analysis of trace elements in solution.

The dissolved-element content was measured using inductively coupled plasma-optical emission spectroscopy (ICP-OES) (Spectro, Varian Vista AX) with Ar as the carrier gas. The analytical error was ∼4%. All measurements were obtained using multielement standards for inductively coupled plasma-mass spectroscopy ICP-MS (LGC Promochem).

### DNA extraction.

Triplicate samples of genomic DNA were extracted from each sample site using a FastDNA soil kit from MP Biomedicals (Qbiogene, Illkrich, France) and an MP Biomedicals homogenizer (speed 5.5 for 40 s) according to the manufacturer's instructions. An additional washing step using 5.5 M guanidine thiocyanate was performed in accordance with a protocol provided by the manufacturer. Genomic DNA of pure archaeal and bacterial cultures was recovered using a DNeasy blood and tissue kit (Qiagen, Hilden, Germany).

### Construction of amplicon libraries for Illumina sequencing.

The 16S rRNA amplicon libraries were constructed in triplicate as described elsewhere (61) and subsequently pooled in equimolar amounts. Sequencing was performed at SciLifeLab, Stockholm, Sweden, using a MiSeq Illumina sequencing platform. Preparation for Illumina sequencing was performed using triplicate DNA extractions and using a two-step PCR approach (61). The first PCR was conducted with the primers 515F (GTGBCAGCMGCCGCGGTAA) and 805R (GGACTACHVGGGTWTCTAAT) (62) to amplify the V4 region of both bacterial and archaeal 16S rRNA genes. Due to extraction and primer biases, our results are all relative abundances.

### Sequence data analysis.

Sequence analyses were performed as described earlier (61). In brief, the sequences were first quality trimmed and further processed using the Quantitative Insights into Microbial Ecology (QIIME) software package, version 1.8 (63). The sequence data were clustered into OTUs at 97% sequence similarity using an open reference OTU picking strategy. The most abundant sequences in each OTU were selected as representative sequences and further aligned against the Greengenes core set using PyNAST software (63). Taxonomy was assigned to each OTU using the Ribosomal Database Project (RDP) classifier with a minimum confidence threshold of 80% (64). The chimeric sequences were removed by ChimeraSlayer (65), and the final OTU table was filtered based on the criterion that OTUs observed in all three replicates were retained. The OTU tables were subsampled (according to the sample containing the smallest set of sequences) to equalize the sampling depths. Bacterial sequences were separated from the total sequence set and retained for further analysis.

### Accession number(s).

The sequences determined in this study have been deposited in the NCBI Sequence Read Archive (SRA) under accession number PRJNA353093.

## Supplementary Material

Supplemental material

## References

[1] Tiago I, Veríssimo A 2013 Microbial and functional diversity of a subterrestrial high pH groundwater associated to serpentinization. Environ Microbiol 15:1687–1706. doi:10.1111/1462-2920.12034. 23731249

[2] Bolhuis H, Cretoiu MS, Stal LJ 2014 Molecular ecology of microbial mats. FEMS Microbiol Ecol 90:335–350. doi:10.1111/1574-6941.12408. 25109247

[3] McCollom TM, Seewald JS 2007 Geochemical constraints on sources of metabolic energy for chemolithoautotrophy in ultramafic-hosted deep-sea hydrothermal systems. Astrobiology 7:933–950. doi:10.1089/ast.2006.0119. 18163871

[4] Chivian D, Brodie EL, Alm EJ, Culley DE, Dehal PS, DeSantis TZ, Gihring TM, Lapidus A, Lin LH, Lowry SR, Moser DP, Richardson PM, Southam G, Wanger G, Pratt LM, Andersen GL, Hazen TC, Brockman FJ, Arkin AP, Onstott TC 2008 Environmental genomics reveals a single-species ecosystem deep within earth. Science 322:275–278. doi:10.1126/science.1155495. 18845759

[5] Proskurowski G, Lilley MD, Seewald JS, Früh-Green GL, Olson EJ, Lupton JE, Sylva SP, Kelley DS 2008 Abiogenic hydrocarbon production at lost city hydrothermal field. Science 319:604–607. doi:10.1126/science.1151194. 18239121

[6] Suzuki S, Ishii S, Wu A, Cheung A, Tenney A, Wanger G, Kuenen Gijs J, Nealson KH 2013 Microbial diversity in The Cedars, an ultrabasic, ultrareducing, and low salinity serpentinizing ecosystem. Proc Natl Acad Sci U S A 110:15336–15341. doi:10.1073/pnas.1302426110. 24003156 PMC3780913

[7] Frost BR, Beard JS 2007 On silica activity and serpentinization. J Petrol 48:1351–1368. doi:10.1093/petrology/egm021.

[8] Sleep NH, Meibom A, Fridriksson T, Coleman RG, Bird DK 2004 H2-rich fluids from serpentinization: geochemical and biotic implications. Proc Natl Acad Sci U S A 101:12818–12823. doi:10.1073/pnas.0405289101. 15326313 PMC516479

[9] Takai K, Inagaki F, Horikoshi K 2004 Distribution of unusual archaea in subsurface biosphere. Geophys Monogr Ser 144:369–381.

[10] Brazelton WJ, Nelson B, Schrenk MO 2011 Metagenomic evidence for H_2_ oxidation and H_2_ production by serpentinite-hosted subsurface microbial communities. Front Microbiol 2:268. doi:10.3389/fmicb.2011.00268. PMC3252642 22232619

[11] McCollom TM 1999 Methanogenesis as a potential source of chemical energy for primary biomass production by autotrophic organisms in hydrothermal systems on Europa. J Geophys Res Planets 104:30729–30742. doi:10.1029/1999JE001126.

[12] McCollom TM, Simoneit BRT 1999 Abiotic formation of hydrocarbons and oxygenated compounds during thermal decomposition of iron oxalate. Orig Life Evol Biosph 29:167–186. doi:10.1023/A:1006556315895. 10227202

[13] Rushdi A, Simoneit BRT 2001 Lipid formation by aqueous Fischer-Tropsch-type synthesis over a temperature range of 100 to 400 degrees C. Orig Life Evol Biosph 31:103–118. doi:10.1023/A:1006702503954. 11296515

[14] Rushdi AI, Simoneit BRT 2005 Abiotic synthesis of organic compounds from carbon disulfide under hydrothermal conditions. Astrobiology 5:749–769. doi:10.1089/ast.2005.5.749. 16379529

[15] Seewald JS, Zolotov MY, McCollom T 2006 Experimental investigation of single carbon compounds under hydrothermal conditions. Geochim Cosmochim Acta 70:446–460. doi:10.1016/j.gca.2005.09.002.

[16] Etiope G, Schoell M, Hosgörmez H 2011 Abiotic methane flux from the Chimaera seep and Tekirova ophiolites (Turkey): understanding gas exhalation from low temperature serpentinization and implications for Mars. Earth Planet Sci Lett 310:96–104. doi:10.1016/j.epsl.2011.08.001.

[17] Hosgörmez H, Etiope G, Yalcin MN 2008 New evidence for a mixed inorganic and organic origin of the Olympic Chimaera fire (Turkey): a large onshore seepage of abiogenic gas. Geofluids 8:263–273. doi:10.1111/j.1468-8123.2008.00226.x.

[18] Etiope G, Schoell M 2014 Abiotic gas: atypical, but not rare. Elements 10:291–296. doi:10.2113/gselements.10.4.291.

[19] Woodcock NH, Robertson AHF 1977 Origins of some ophiolite-related metamorphic rocks of the “Tethyan” belt. Geology 5:373. doi:10.1130/0091-7613(1977)5<373:OOSOMR>2.0.CO;2.

[20] Meyer-Dombard DR, Woycheese KM, Yargiçoğul, Cardace D, Shock EL, Güleçal-Pektas Y, Temel M 2014 High pH microbial ecosystems in a newly discovered, ephemeral, serpentinizing fluid seep at Yanartaş (Chimera), Turkey. Front Microbiol 5:723. doi:10.3389/fmicb.2014.00723. 25646094 PMC4298219

[21] Hosgörmez H 2007 Origin of the natural gas seep of Cirali (Chimera), Turkey: site of the first Olympic fire. J Asian Earth Sci 30:131–141. doi:10.1016/j.jseaes.2006.08.002.

[22] Brazelton WJ, Schrenk MO, Kelley DS, Baross JA 2006 Methane- and sulfur-metabolizing microbial communities dominate the lost city hydrothermal field ecosystem. Appl Environ Microbiol 72:6257–6270. doi:10.1128/AEM.00574-06. 16957253 PMC1563643

[23] Cui M, Ma A, Qi H, Ahuzng X, Zhuang G 2015 Anaerobic oxidation of methane: an “active” microbial process. Microbiologyopen 4:1–11. (Geol Rundsch) doi:10.1002/mbo3.232. 25530008 PMC4335971

[24] Bağci U, Parlak O 2007 Petrology of the Tekirova (Antalya) ophiolite (Southern Turkey): evidence for diverse magma generations and their tectonic implications during Neotethyan-subduction. Int J Earth Sci 98:387–405.

[25] Aldanmaz E, Schmidt MW, Gourgaud A, Meisel T 2009 Mid-ocean ridge and supra-subduction geochemical signatures in spinel—peridotites from the Neotethyan ophiolites in SW Turkey: implications for upper mantle melting processes. Lithos 113:691–708. doi:10.1016/j.lithos.2009.03.010.

[26] Demirel IH, Gunay Y 2000 Tectonic and karstic effects on the western Taurus region, southwestern Turkey: relations to the present temperature gradients and total organic carbon content. Energy Sources 22:431–441. doi:10.1080/00908310050013848.

[27] Chavagnac V, Ceuleneer G, Monnin C 2013 Mineralogical assemblages forming at hyperalkaline warm springs hosted on ultramafic rocks: a case study of Oman and Ligurian ophiolites. Geochemistry 14:2474–2495.

[28] Neal C, Stanger G 1984 Calcium and magnesium hydroxide precipitation from alkaline groundwaters in Oman, and their significance to the process of serpentinization. Mineral Mag 48:237–241. doi:10.1180/minmag.1984.048.347.07.

[29] Pedersen K, Nilsson E, Arlinger J, Hallbeck L, O'Neill A 2004 Distribution, diversity and activity of microorganisms in the hyper-alkaline spring waters of Maqarin in Jordan. Extremophiles 8:151–164. doi:10.1007/s00792-004-0374-7. 14991423

[30] Veríssimo A, Tiago I 2015 Bacterial diversity in a nonsaline alkaline environment, p 37–41. *In* Nelson KE (ed), Encyclopedia of metagenomics. Springer, Boston, MA.

[31] Barnes I, LaMarche VC, Himmelberg G 1967 Geochemical evidence of present-day serpentinization. Science 156:830–832. doi:10.1126/science.156.3776.830. 17780302

[32] Neal C, Shand P 2002 Spring and surface water quality of the Cyprus ophiolites. Hydrol Earth Syst Sci 6:797–817. doi:10.5194/hess-6-797-2002.

[33] Früh-Green GL, Connolly JAD, Plas A, Kelley DS, Grobety B 2004 Serpentinization of oceanic peridotites: implications for geochemical cycles and biological activity, p 119–136. *In* Wilcock WS, Delong EF, Kelley DS, Baross JA, Cary SC (ed), The subseafloor biosphere at mid-ocean ridges. American Geophysical Union, Washington, DC.

[34] Yoon JH, Kang SJ, Ryu SH, Jeon CO, Oh TK 2008 Hydrogenophaga bisanensis sp. nov., isolated from wastewater of a textile dye works. Int J Syst Evol Microbiol 58:393–397. doi:10.1099/ijs.0.65271-0. 18218937

[35] Kim Y-J, Kim MK, Weon H-Y, Kim H-B, Yang D-C 2010 Hydrogenophaga temperata sp. nov., a betaproteobacterium isolated from compost in Korea. J Gen Appl Microbiol 56:419–425. doi:10.2323/jgam.56.419. 21282897

[36] Roadcap G, Bethke CM, Sanford RA 2003 Microbial community found thriving in very alkaline (pH 12-13) groundwater, abstr 143-9. Abstr Annu Meet Geol Soc Am. Geological Society of America, Washington, DC.

[37] Faure G 1998 Principles and applications of geochemistry: a comprehensive textbook for geology students. Prentice Hall, Upper Saddle River, NJ.

[38] Hug LA, Castelle CJ, Wrighton KC, Thomas BC, Sharon I, Frischkorn KR, Williams KH, Tringe SG, Banfield JF 2013 Community genomic analyses constrain the distribution of metabolic traits across the Chloroflexi phylum and indicate roles in sediment carbon cycling. Microbiome 1:22. doi:10.1186/2049-2618-1-22. 24450983 PMC3971608

[39] Barton HA, Giarrizzo JG, Suarez P, Robertson CE, Broering MJ, Banks ED, Vaishampayan PA, Venkateswaran K 2014 Microbial diversity in a Venezuelan orthoquartzite cave is dominated by the *Chloroflexi* (class *Ktedonobacterales*) and *Thaumarchaeota* group I.1c. Front Microbiol 5:615. doi:10.3389/fmicb.2014.00615. 25505450 PMC4244709

[40] Engel AS, Meisinger DB, Porter ML, Payn RA, Schmid M, Stern LA, Schleifer KH, Lee NM 2010 Linking phylogenetic and functional diversity to nutrient spiraling in microbial mats from Lower Kane Cave (USA). ISME J 4:98–110. doi:10.1038/ismej.2009.91. 19675595

[41] Lehtoranta J, Heiskanen A-S 2003 Dissolved iron:phosphate ratio as an indicator of phosphate release to oxic water of the inner and outer coastal Baltic Sea. Hydrobiologia 492:69–84. doi:10.1023/A:1024822013580.

[42] Sonia M-T, Naceur J, Abdennaceur H 2011 Studies on the ecology of actinomycetes in an agricultural soil amended with organic residues: I. identification of the dominant groups of *Actinomycetales*. World J Microbiol Biotechnol 27:2239–2249. doi:10.1007/s11274-011-0687-5.

[43] Quaiser A, Ochsenreiter T, Lanz C 2003 Acidobacteria form a coherent but highly diverse group within the bacterial domain: evidence from environmental genomics. Mol Microbiol 50:563–575. doi:10.1046/j.1365-2958.2003.03707.x. 14617179

[44] Fierer N, Bradford MA, Jackson RB 2007 Toward an ecological classification of soil bacteria. Ecology 88:1354–1364. doi:10.1890/05-1839. 17601128

[45] Gagliano AL, Tagliavia M, D'Alessandro W, Franzetti A, Parello F, Quatrini P 2016 So close, so different: geothermal flux shapes divergent soil microbial communities at neighbouring sites. Geobiology 14:150–162. doi:10.1111/gbi.12167. 26560641

[46] Quéméneur M, Palvadeau A, Postec A, Monnin C, Chavagnac V, Ollivier B, Erauso G 2015 Endolithic microbial communities in carbonate precipitates from serpentinite-hosted hyperalkaline springs of the Voltri Massif (Ligurian Alps, Northern Italy). Environ Sci Pollut Res Int 22:13613–13624. doi:10.1007/s11356-015-4113-7. 25874424

[47] Chen J 2012 Statistical methods for human microbiome data analysis. PhD dissertation. University of Pennsylvania, Philadelphia, PA.

[48] Sutton NB, van der Kraan GM, van Loosdrecht MC, Muyzer G, Bruining J, Schotting RJ 2009 Characterization of geochemical constituents and bacterial populations associated with As mobilization in deep and shallow tube wells in Bangladesh. Water Res 43:1720–1730. doi:10.1016/j.watres.2009.01.006. 19215956

[49] Li X, Bond PL, Van Nostrand JD, Zhou J, Huang L 2015 From lithotroph-to organotroph-dominant: directional shift of microbial community in sulphidic tailings during phytostabilization. Sci Rep 5:12978. doi:10.1038/srep12978. 26268667 PMC4534789

[50] Xia Y, Wang Y, Wang Y, Chin FYL, Zhang T 2016 Cellular adhesiveness and cellulolytic capacity in Anaerolineae revealed by omics-based genome interpretation. Biotechnol Biofuels 9:111. doi:10.1186/s13068-016-0524-z. 27222666 PMC4877987

[51] Zheng C, Yu L, Huang L, Xiu J, Huang Z 2012 Investigation of a hydrocarbon-degrading strain, *Rhodococcus ruber* Z25, for the potential of microbial enhanced oil recovery. J Pet Sci Eng 81:49–56. doi:10.1016/j.petrol.2011.12.019.

[52] Fukunaga Y, Kurahashi M, Sakiyama Y, Ohuchi M, Yokota A, Harayama S 2009 *Phycisphaera mikurensis* gen. nov., sp. nov., isolated from a marine alga, and proposal of *Phycisphaeraceae* fam. nov., *Phycisphaerales* ord. nov and *Phycisphaerae* classis nov in the phylum *Planctomycetes*. J Gen Appl Microbiol 55:267–275. doi:10.2323/jgam.55.267. 19700920

[53] Bodrossy L, Holmes EM, Holmes AJ, Kovács KL, Murrell JC 1997 Analysis of 16S rRNA and methane monooxygenase gene sequences reveals a novel group of thermotolerant and thermophilic methanotrophs, *Methylocaldum* gen. nov. Arch Microbiol 168:493–503. doi:10.1007/s002030050527. 9385141

[54] Takeuchi M, Kamagata Y, Oshima K, Hanada S, Tamaki H, Marumo K, Maeda H, Nedachi M, Hattori M, Iwasaki W, Sakata S 2014 *Methylocaldum marinum* sp. nov., a thermotolerant, methane-oxidizing bacterium isolated from marine sediments, and emended description of the genus *Methylocaldum*. Int J Syst Evol Microbiol 64:3240–3246. doi:10.1099/ijs.0.063503-0. 24981325

[55] Ghiorse WC 1984 Biology of iron- and manganese-depositing bacteria. Annu Rev Microbiol 38:515–550. doi:10.1146/annurev.mi.38.100184.002503. 6388499

[56] Stoecker K, Bendinger B, Schöning BR, Nielsen PH, Nielsen JL, Baranyi C, Toenshoff ER, Daims H, Wagner M 2006 Cohn's *Crenothrix* is a filamentous methane oxidizer with an unusual methane monooxygenase. Proc Natl Acad Sci U S A 103:2363–2367. doi:10.1073/pnas.0506361103. 16452171 PMC1413686

[57] Normand P 2006 *Geodermatophilaceae* fam. nov., a formal description. Int J Syst Evol Microbiol 56:2277–2278. doi:10.1099/ijs.0.64298-0. 17012547

[58] Lopes AR, Manaia CM, Nunes OC 2014 Bacterial community variations in an alfalfa-rice rotation system revealed by 16S rRNA gene 454-pyrosequencing. FEMS Microbiol Ecol 87:650–663. doi:10.1111/1574-6941.12253. 24245591

[59] Downs RT 2006 The RRUFF Project: an integrated study of the chemistry, crystallography, Raman and infrared spectroscopy of minerals, p 3–13. *In* Program and abstracts of the 19th general meeting of the International Mineralogical Association, Kobe, Japan.

[60] Dawson P, Hadfield CD, Wilkinson GR 1973 The polarized infra-red and Raman spectra of Mg(OH)_2_ and Ca(OH)_2_. J Physics Chem Solids 34:1217–1225. doi:10.1016/S0022-3697(73)80212-4.

[61] Müller B, Sun L, Westerholm M, Schnürer A 2016 Bacterial community composition and *fhs* profiles of low- and high-ammonia biogas digesters reveal novel syntrophic acetate-oxidising bacteria. Biotechnol Biofuels 9:48. doi:10.1186/s13068-016-0454-9. 26925165 PMC4769498

[62] Hugerth LW, Muller E, Hu YO, Lebrun LA, Roume H, Lundin D, Wilmes P, Andersson AF 2014 Systematic design of 18S rRNA gene primers for determining eukaryotic diversity in microbial consortia. PLoS One 9:e95567. doi:10.1371/journal.pone.0095567. 24755918 PMC3995771

[63] Caporaso JG, Bittinger K, Bushman FD, DeSantis TZ, Andersen GL, Knight R 2010 PyNAST: a flexible tool for aligning sequences to a template alignment. Bioinformatics 26:266–267. doi:10.1093/bioinformatics/btp636. 19914921 PMC2804299

[64] Wang Q, Garrity GM, Tiedje JM, Cole JR 2007 Naïve Bayesian classifier for rapid assignment of rRNA sequences into the new bacterial taxonomy. Appl Environ Microbiol 73:5261–5267. doi:10.1128/AEM.00062-07. 17586664 PMC1950982

[65] Haas BJ, Gevers D, Earl AM, Feldgarden M, Ward DV, Giannoukos G, Ciulla D, Tabbaa D, Highlander SK, Sodergren E, Methe B, DeSantis TZ, Human Microbiome Consortium, Petrosino JF, Knight R, Birren BW 2011 Chimeric 16S rRNA sequence formation and detection in Sanger and 454-pyrosequenced PCR amplicons. Genome Res 21:494–504. doi:10.1101/gr.112730.110. 21212162 PMC3044863

