# Supplementary material for: Microbial Community Structure in a Serpentine-Hosted Abiotic Gas Seepage at the Chimaera Ophiolite, Turkey
Source: Appl Environ Microbiol. 2017 May 31;83(12):e03430-16. doi: 10.1128/AEM.03430-16 (PMC5452829; doi:10.1128/AEM.03430-16)
Supplement: Supplemental material [file supp_83_12_e03430-16__index.html]

Supplemental material 

# Microbial Community Structure in a Serpentine-Hosted Abiotic Gas Seepage at the Chimaera Ophiolite, Turkey

## Supplemental material

- Supplemental file 1 -

  Simplified geological map of the study area and location (Fig. S1), images of the sampling sites (Fig. S2), major and minor dissolved elements measured with ICP-OES (Table S1), metadata used for UniFrac and CCA (Table S2), rarefaction analysis of bacterial communities at Chimaera ophiolite (Fig. S3), relative percent distribution of classes of *Proteobacteria* in all samples (Fig. S4), relative percent distribution of orders of bacteria and archaea in all samples (Table S3), and CCA plots showing correlation between OTUs and geochemical metadata (Fig. S5 and S6).

  PDF, 3.0M
