# Supplementary material for: Microbial Community Structure in a Serpentine-Hosted Abiotic Gas Seepage at the Chimaera Ophiolite, Turkey
Source: Appl Environ Microbiol. 2017 May 31;83(12):e03430-16. doi: 10.1128/AEM.03430-16 (PMC5452829; doi:10.1128/AEM.03430-16)
Supplement: Supplemental material [file AEM.03430-16_zam999117866s1.pdf]

Supplementary information

Microbial community structure of an abiotic serpentine-hosted gas seepage at the Chimaera ophiolite, Turkey

Anna Neubeck[1*], Li Sun[2], Bettina Müller[2], Magnus Ivarsson[3], Hakan Hosgörmez[4], Dogacan Özcan[4], Curt Broman[1], Anna Schnürer[2]

[1]Department of Geological Sciences, Stockholm University, Sweden, Svante Arrhenius väg 8, 10691 Stockholm; Phone: +468164747, Fax: 46 8 674 78 97; Email: anna.neubeck@geo.su.se*

[2]Department of Microbiology, Swedish University of Agricultural Sciences, Uppsala, Sweden

[3]Swedish Museum of Natural History, Department of Paleobiology and Nordic Center for Earth Evolution (NordCEE), Box 50007, SE-105 04 Stockholm, Sweden.

[4]Department of Geological Engineering, Istanbul University, Istanbul, Turkey

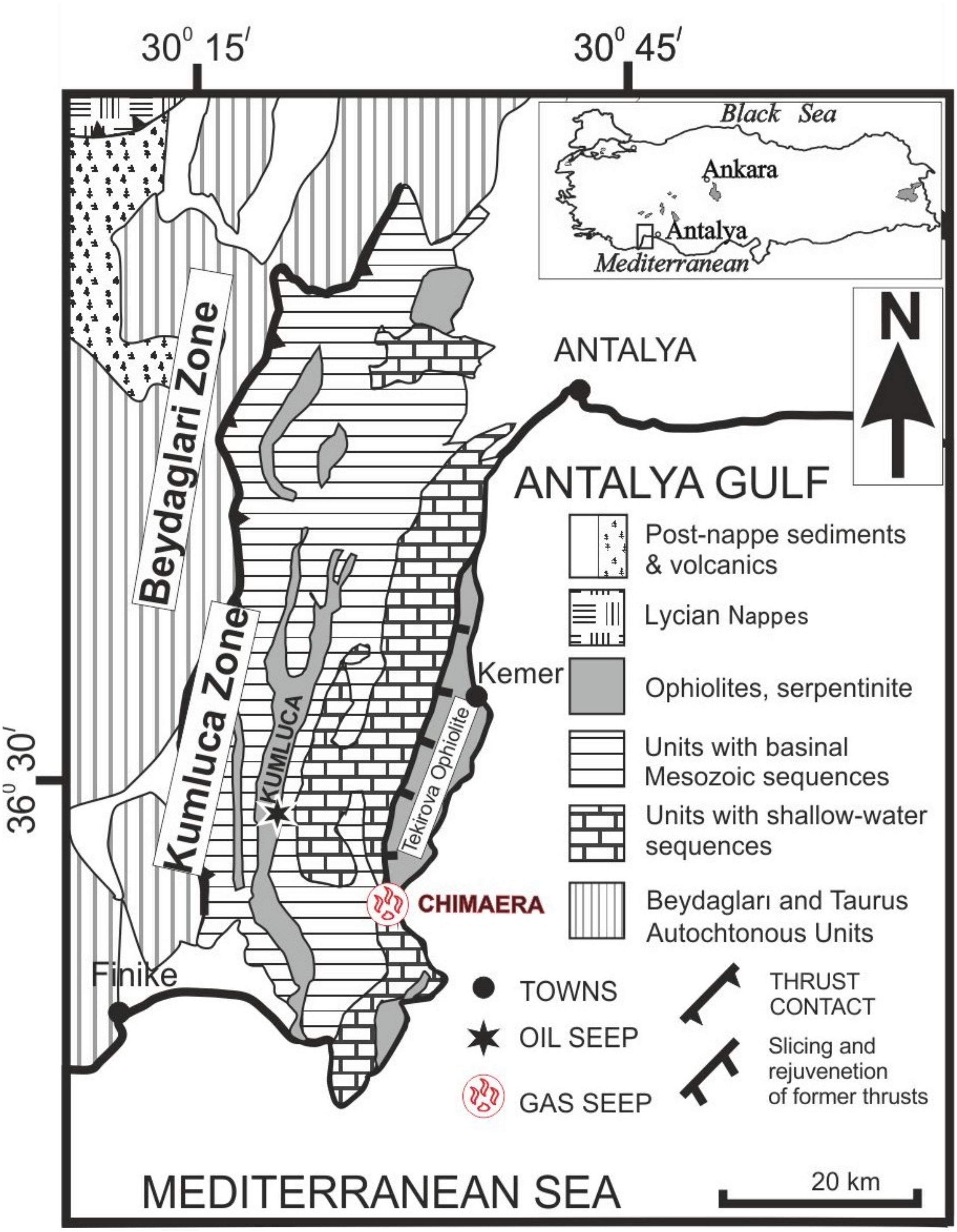

Figure S1. Simplified geological map of the study area and location. Modified after Robertson and Woodcock (1980) and (Hosgormez et al., 2008)

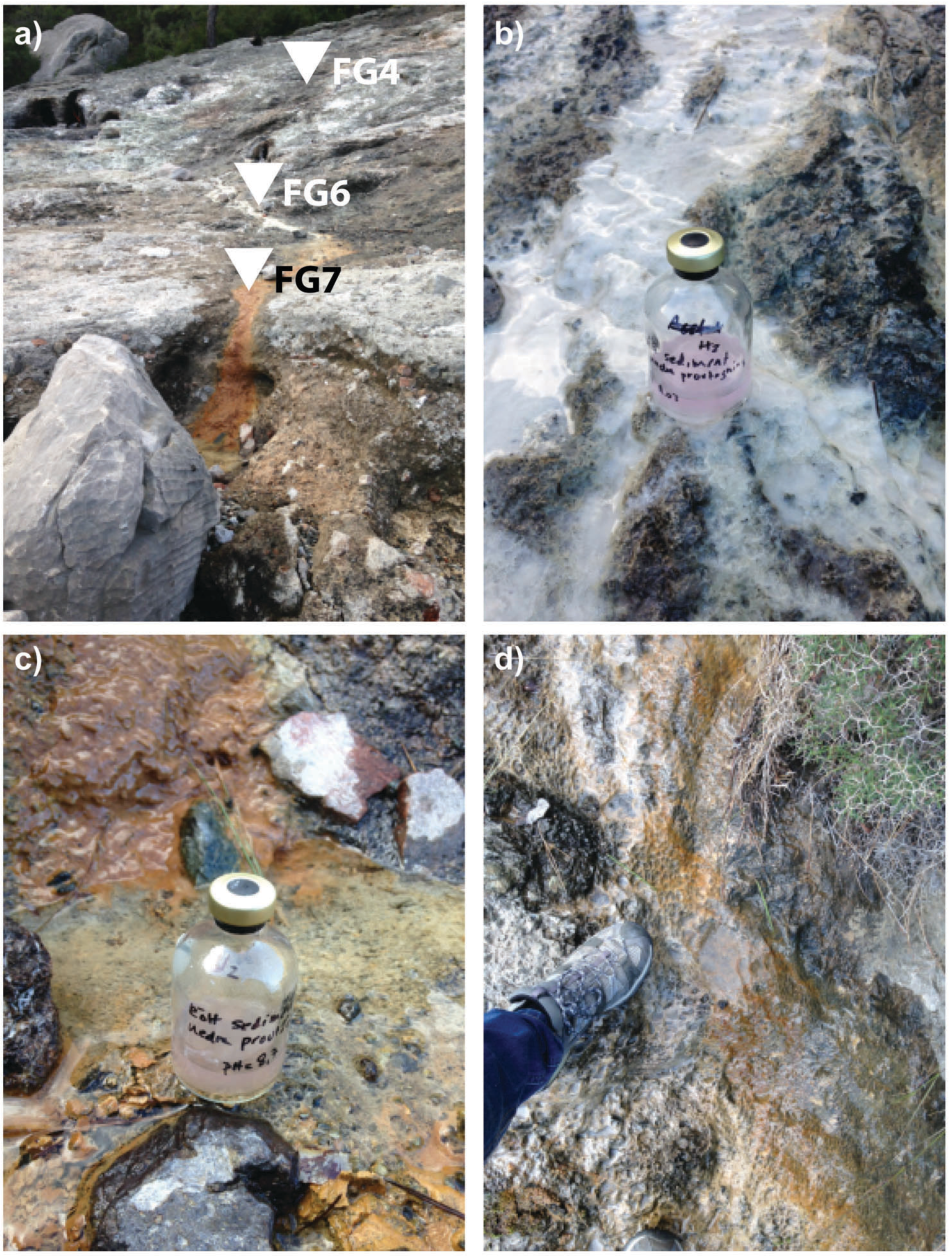

Figure S2. Images of the sampling sites showing a) the FG rill viewed uphill where FG4, FG6 and FG7 is marked out, b) FG6, c) FG7 (similar to IR site), d) BF site consisting of biofilm-covered travertines.

Table S1. Major and minor dissolved elements measured with ICP-OES (ppb).The analytical error is ~4%. Cells marked with <DL means below detection limit. Samples marked with (sed) had sediments in the sample tubes.

Major elements (ppb)						
**Sample**	**Mg**	**Ca**	**Na**	**S**	**Si**	**Al**
BF1	9,50E+00	2,40E+01	7,20E+00	8,30E+00	4,90E+00	1,40E-02
BF2	8,80E+00	1,80E+01	7,30E+00	8,00E+00	5,00E+00	2,90E-02
FG3	1,00E+02	5,80E+01	1,20E+01	9,40E+00	4,20E+01	3,70E-01
FG4	1,20E+02	3,20E+01	1,40E+01	1,70E+01	3,60E+01	4,60E-01
FG5	8,50E+01	5,00E+01	1,20E+01	1,10E+01	3,70E+01	8,10E-03
FG6	3,20E+01	7,40E+01	1,20E+01	1,80E+01	5,70E+00	3,70E-02
FG6	4,20E+01	5,40E+01	1,30E+01	1,50E+01	7,20E+00	1,90E-02
FG7.1	8,90E+01	3,30E+01	1,30E+01	2,30E+01	2,90E+01	1,10E-02
FG7.2	9,20E+01	4,10E+01	1,20E+01	1,40E+01	2,90E+01	6,20E-02
IR	9,20E+01	3,00E+01	7,90E+00	8,10E+00	3,20E+01	2,30E-02
JSB	7,70E+01	2,20E+01	8,40E+00	8,30E+00	2,30E+01	2,80E-02
LB	8,20E+01	6,30E+01	1,20E+01	1,80E+01	3,70E+01	1,10E-01
LB1	1,20E+02	7,00E+01	2,10E+01	8,90E+00	5,80E+01	1,20E+01
LB2	8,70E+01	5,20E+01	1,20E+01	1,70E+01	3,50E+01	1,60E-02
**Minor elements (ppb)**						
**Sample**	**As**	**Ba**	**Cr**	**Cu**	**Fe**	**K**
BF1	2,00E-02	6,80E-03	5,60E-03	6,20E-03	2,70E-02	7,10E-01
BF2	1,50E-02	6,10E-03	5,80E-03	3,90E-03	1,60E-01	6,70E-01
FG3	1,40E-02	7,80E-03	3,00E-02	9,50E-03	1,50E+00	6,20E-01
FG4	1,90E-02	1,00E-02	3,60E-02	8,30E-03	1,60E+00	1,30E+00
FG5	1,50E-02	5,10E-03	1,20E-03	4,80E-03	1,00E-02	4,90E-01
FG6	2,40E-02	7,00E-03	1,50E-03	1,10E-02	5,60E-02	6,70E-01
FG6	1,40E-02	7,20E-03	1,20E-03	3,10E-02	6,40E-02	6,70E-01
FG7.1	2,40E-02	5,30E-03	1,10E-03	4,50E-03	1,60E-02	7,00E-01
FG7.2	1,20E-02	6,10E-03	1,40E-02	9,50E-03	8,40E-01	7,20E-01
IR	1,90E-02	6,20E-03	4,30E-03	7,10E-03	5,60E-02	6,30E-01
JSB	1,70E-02	6,00E-03	2,90E-03	1,10E-02	3,90E-02	7,60E-01
LB	2,30E-02	7,00E-02	8,60E-03	6,20E-03	4,50E-01	5,70E-01
LB1	1,20E-02	3,80E-02	1,80E-01	1,80E-02	9,50E+00	6,10E-01
LB2	1,70E-02	6,40E-03	1,90E-03	4,90E-03	1,10E-02	5,10E-01
**Minor elements (ppb)**						
**Sample**	**Mn**	**Ni**	**P**	**Sr**	**Ti**	**Zn**
BF1	1,10E-03	5,40E-03	2,40E-02	8,20E-02	2,10E-03	1,00E-02
BF2	7,80E-03	1,10E-02	1,20E-01	7,00E-02	1,70E-03	2,40E-02
FG3	1,40E-02	7,90E-02	2,30E-02	8,30E-02	1,10E-02	2,50E-02
FG4	9,70E-02	1,30E-01	2,80E-01	4,60E-02	1,20E-02	1,90E-02

FG5	1,30E-03	6,70E-03	7,60E-03	7,10E-02	1,30E-03	8,40E-03
FG6	2,00E-03	3,00E-03	1,70E-02	1,20E-01	4,80E-03	1,60E-02
FG6	1,50E-03	4,30E-03	1,90E-02	9,30E-02	1,40E-03	3,00E-02
FG7.1	7,50E-04	6,80E-03	1,10E-02	5,00E-02	1,30E-03	1,00E-02
FG7.2	1,60E-02	4,50E-02	1,60E-01	7,00E-02	3,30E-03	3,30E-02
IR	2,80E-03	4,90E-03	6,40E-02	4,80E-02	1,70E-03	1,60E-02
JSB	2,00E-03	3,60E-03	2,50E-02	3,90E-02	1,80E-03	2,00E-02
LB	1,80E-02	4,50E-02	2,80E-02	8,40E-02	2,70E-03	2,20E-02
LB1	2,90E-01	4,90E-01	1,40E-01	9,80E-02	8,00E-02	3,20E-02
LB2	1,60E-03	8,00E-03	1,90E-02	7,20E-02	1,30E-03	1,30E-02
**Minor elements (ppb)**						
**Sample**	**Be**	**Co**	**Li**	**Mo**	**V**	
BF1	1,20E-05	4,60E-04	<DL	8,60E-04	<DL	
BF2	<DL	5,30E-04	<DL	<DL	<DL	
FG3	2,90E-05	2,90E-03	1,20E-03	7,10E-04	1,10E-03	
FG4	<DL	6,40E-03	1,30E-03	9,30E-04	4,20E-03	
FG5	2,30E-05	<DL	1,10E-03	9,30E-04	7,50E-04	
FG6	2,40E-05	<DL	6,60E-04	<DL	<DL	
FG6	<DL	<DL	6,90E-04	<DL	<DL	
FG7.1	2,10E-05	<DL	9,10E-04	<DL	7,10E-04	
FG7.2	2,00E-05	2,30E-03	9,90E-04	7,30E-04	1,30E-03	
IR	1,20E-05	<DL	6,10E-04	<DL	<DL	
JSB	1,40E-05	<DL	6,30E-04	<DL	<DL	
LB	1,50E-05	1,70E-03	1,10E-03	<DL	1,80E-03	
LB1	3,20E-05	2,60E-02	1,70E-03	<DL	1,50E-02	
LB2	1,60E-05	<DL	1,00E-03	<DL	9,60E-04	

Table S2. Metadata used for UniFrac and CCA.

Sites	Al	As	B_	Ba	Ca	Cd	Co	Cr	Cu
BF1	13,8	20,2	0	6,8	23737,7	0	0,5	5,6	6,2
BF2	28,9	15,4	48	6,1	17821,1	0	0,5	5,8	3,9
FG4	457,2	18,7	456,9	10,1	31599	0	6,4	36,1	8,3
FG6	18,8	14,1	22,1	7,2	54423,3	0	0	1,2	31,2
FG7.1	10,9	24,2	0	5,3	33087,7	0	0	1,1	4,5
FG7.2	10,9	24,2	0	5,3	33087,7	0	0	1,1	4,5
FG7.3	10,9	24,2	0	5,3	33087,7	0	0	1,1	4,5
IR	23	18,8	5,3	6,2	29665,6	0	0	4,3	7,1
JSB	27,6	16,6	20,4	6	22344,7	0	0	2,9	10,8
LB	111,7	23,1	110	70,4	63183	0	1,7	8,6	6,2
LB1	11856,9	12	2513,2	37,9	70474,4	0,5	26	178,7	18

Sites	Fe	K_	Li	Mg	Mn	Mo	Na	Ni	P_
BF1	27,5	711,2	0	9484,9	1,1	0,9	7161,2	5,4	24,2
BF2	157,9	672,1	0	8847,8	7,8	0	7323,7	10,7	123,3
FG4	1631,7	1347,7	1,3	116424,1	96,7	0,9	13943,4	133,4	282,3
FG6	64,4	666,5	0,7	41652,7	1,5	0	12628,9	4,3	19
FG7.1	15,8	698,3	0,9	89361,3	0,8	0	12709,3	6,8	10,7
FG7.2	15,8	698,3	0,9	89361,3	0,8	0	12709,3	6,8	10,7
FG7.3	15,8	698,3	0,9	89361,3	0,8	0	12709,3	6,8	10,7
IR	55,8	629,5	0,6	92480,6	2,8	0	7888,8	4,9	64,2
JSB	39	756,5	0,6	77091,3	2	0	8367,5	3,6	25,2
LB	446	573,7	1,1	81930,7	17,9	0	11654,4	45,1	27,7
LB1	9490	607,5	1,7	117140,8	288,6	0	21082,4	485,4	140,3

Sites	Pb	S_	Si	Sr	Ti	V_	Zn	pH
BF1	0	8341,2	4867,1	82	2,1	0	10,1	11,4
BF2	0	7998,2	4969,9	69,7	1,7	0	24	11,4
FG4	2,4	17233,9	35909,6	46,3	11,7	4,2	19,1	9
FG6	2,6	15281,3	7233,4	93,5	1,4	0	29,7	10,7
FG7.1	0	23145,7	28771,2	50,4	1,3	0,7	10,3	8,7
FG7.2	0	23145,7	28771,2	50,4	1,3	0,7	10,3	8,7
FG7.3	0	23145,7	28771,2	50,4	1,3	0,7	10,3	8,7
IR	0	8091,8	31862,1	48,4	1,7	0	16,5	7,9
JSB	0	8316,2	23197,9	38,7	1,8	0	19,5	8
LB	0	17885,2	37176,1	84	2,7	1,8	22,1	7,6
LB1	0	8871	58375,5	98,4	80,2	14,6	31,8	7,6

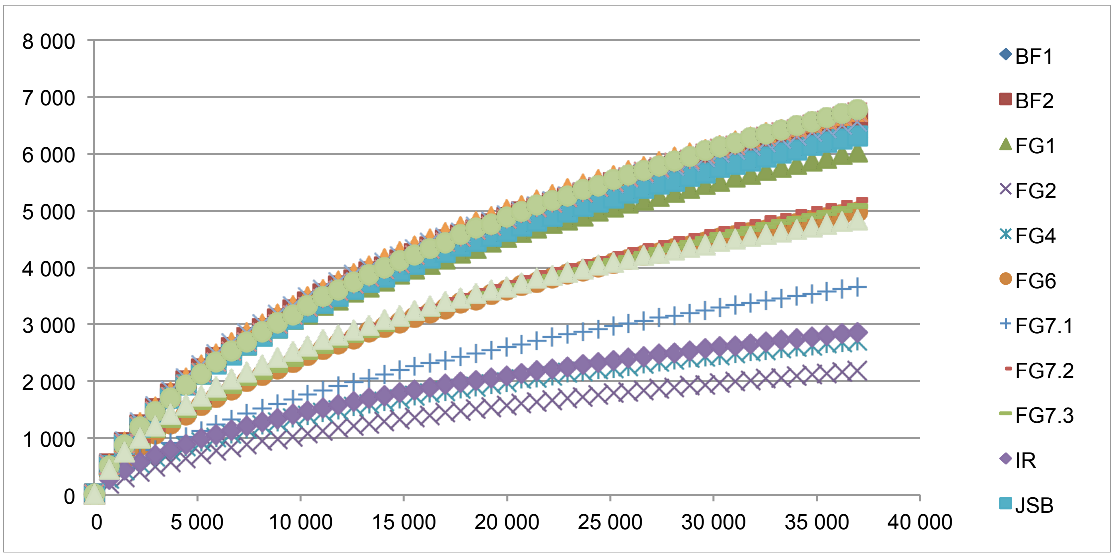

Figure S3. Rarefraction analysis of bacterial communities at Chimaera ophiolite.

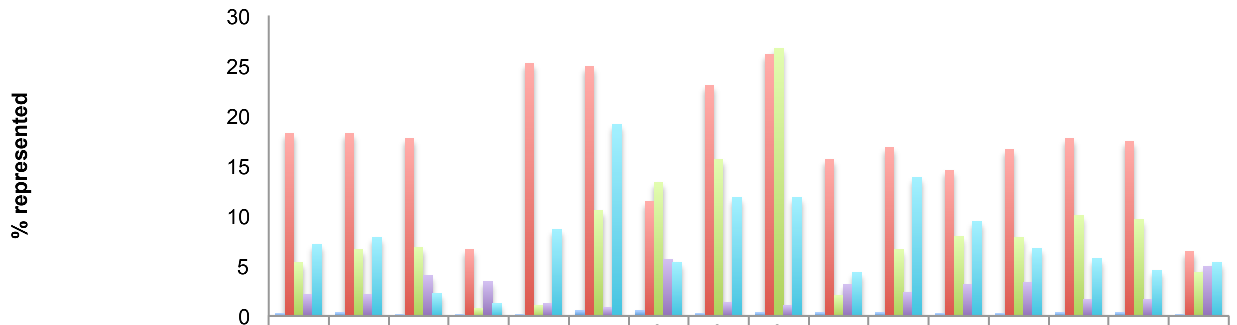

	BF1	BF2	FG1	FG2	FG4	FG6	FG7.1	FG7.2	FG7.3	IR	JSB	LB	LB1	LB2	LB2.2	SB
Other	0.2	0.3	0.1	0.1	0.1	0.5	0.5	0.2	0.3	0.3	0.3	0.2	0.2	0.3	0.3	0.1
Alphaproteobacteria	18.2	18.2	17.7	6.6	25.2	24.9	11.4	23	26.1	15.6	16.8	14.5	16.6	17.7	17.4	6.4
Betaproteobacteria	5.3	6.6	6.8	0.7	1	10.5	13.3	15.6	26.7	2	6.6	7.9	7.8	10	9.6	4.3
Deltaproteobacteria	2.1	2.1	4	3.4	1.2	0.8	5.6	1.3	1	3.1	2.3	3.1	3.3	1.6	1.6	4.9
Gammaproteobacteria	7.1	7.8	2.2	1.2	8.6	19.1	5.3	11.8	11.8	4.3	13.8	9.4	6.7	5.7	4.5	5.3

Fig. S4. Relative percental distribution of classes of Proteobacteria in all samples.

Table. S3. Relative percental distribution of orders of bacteria and archaea in all samples.

Taxonomy	LB	JSB	BF2	BF1	LB1	LB2	FG4	FG6	IR	FG1	FG2	FG7.3	FG7.2	FG7.1	SB	LB2.2
k__Archaea;p__Crenarchaeota;c__Thaumarchaeota;o__Nitrososphaerales	1	0,6	0,6	0,5	0,8	0,1	0	0	0	0,3	0	0,1	0,1	0	12,1	0,1
k__Archaea;p__Euryarchaeota;c__Methanobacteria;o__Methanobacteriales	0	0	0	0	0	0	0	0	0	0	0	0	0,2	0	0	0
k__Bacteria;Other;Other;Other	1,4	4,1	3,4	3	3,8	4,2	4,5	5,5	7,1	1,3	11,6	3,2	5,5	13,6	0,9	4,7
k__Bacteria;p__Acidobacteria;c__;o__	0	0	0	0	0	0	0	0	0	0	0	0	0	0	0,2	0
k__Bacteria;p__Acidobacteria;c__Acidobacteria-6;Other	0,1	0,1	0,1	0,1	0,1	0,1	0	0	0	0,5	0	0	0	0	0,2	0
k__Bacteria;p__Acidobacteria;c__Acidobacteria-6;o__CCU21	0,2	0,1	0	0	0,2	0	0	0	0	0,1	0	0	0	0	0,5	0
k__Bacteria;p__Acidobacteria;c__Acidobacteria-6;o__iii1-15	4,6	2,2	1,4	1,8	4	1,1	0,1	0,2	0,2	4,1	0,1	0,5	0,4	0,1	5,3	1,2
k__Bacteria;p__Acidobacteria;c__Acidobacteriia;o__Acidobacteriales	0,1	0	0	0	0	0	0	0	0	0	0	0	0	0	0,2	0
k__Bacteria;p__Acidobacteria;c__Solibacteres;o__Solibacterales	1,1	1,4	1,1	1,2	1	1,1	0,8	0,2	1,1	1,4	0,6	0,3	0,5	0,9	1,2	1
k__Bacteria;p__Acidobacteria;c__Sva0725;o__Sva0725	0,3	0,2	0,1	0,2	0,2	0	0	0	0	1,3	0	0	0	0	0,2	0
k__Bacteria;p__Acidobacteria;c__[Chloracidobacteria];o__11-24	0,7	0,2	0	0,1	0,3	0,1	0	0	0	0,1	0	0	0	0	0,5	0,1
k__Bacteria;p__Acidobacteria;c__[Chloracidobacteria];o__PK29	0,4	0,5	0,7	0,7	0,4	0,2	0	0	0,1	0,2	0	0	0	0	0,3	0,2
k__Bacteria;p__Acidobacteria;c__[Chloracidobacteria];o__RB41	6,3	5,2	3,8	4,4	4,5	3,6	0,2	1	0,4	2,2	0,2	1,3	1,6	1,7	4,7	3,2
k__Bacteria;p__Acidobacteria;c__iii1-8;o__DS-18	0,6	0,4	0,2	0,2	0,4	0,2	0,1	0,1	0,1	0,5	0,1	0,3	0,2	0	0,8	0,3
k__Bacteria;p__Actinobacteria;Other;Other	0,2	0,1	0,2	0,3	0,2	0,2	0,1	0,1	0	0,3	0,1	0,1	0,1	0,3	0,1	0,2
k__Bacteria;p__Actinobacteria;c__Acidimicrobiia;o__Acidimicrobiales	0,9	0,9	1	1,4	0,8	0,8	0,3	0,6	0,8	1,4	0,3	0,5	0,6	3,8	1,1	0,8
k__Bacteria;p__Actinobacteria;c__Actinobacteria;o__Actinomycetales	7	9	1	12,3	9,3	9,9	11,7	3,3	5,9	11,4	3,6	4,1	5,5	9,4	2,2	7
k__Bacteria;p__Actinobacteria;c__Actinobacteria;o__Micrococcales	0,1	0,3	0,3	0,3	0,1	0,2	0	0,1	0	0,1	0	0,2	0,2	0,2	1	0,1
k__Bacteria;p__Actinobacteria;c__MB-A2-108;o__0319-7L14	0,3	0,2	0,1	0,1	0,2	0,2	0	0	0	0,3	0	0,2	0,1	0,1	1,9	0,2
k__Bacteria;p__Actinobacteria;c__Nitriliruptoria;o__Euzebyales	0	0,3	0,8	1,2	0	0,2	0,3	0,2	0	0,5	0	0,1	0,2	0	0	0,2
k__Bacteria;p__Actinobacteria;c__OPB41;o__	0	0	0	0	0	0	0	0	0	0,2	0	0	0	0	0	0
k__Bacteria;p__Actinobacteria;c__Rubrobacteria;o__Rubrobacterales	2,6	1,7	2,4	2,5	2,2	1,9	0	0,6	0	0,4	0	0,7	0,8	0,1	4,5	1,6
k__Bacteria;p__Actinobacteria;c__Thermoleophilia;o__Gaiellales	1,2	1	1,1	1,3	0,9	1	0,1	0,6	0,4	1	1,2	0,6	0,7	1,3	7,9	0,9
k__Bacteria;p__Actinobacteria;c__Thermoleophilia;o__Solirubrobacterales	3,2	3	4,2	4,8	2,9	3,3	0,5	1,7	1,5	4,9	0,9	1,7	2,2	6,9	2,8	3,2
k__Bacteria;p__Armatimonadetes;c__0319-6E2;o__	0,6	0,4	0,2	0,3	0,4	0,2	0	0	0	0,3	0	0	0,1	0	0,5	0,2
k__Bacteria;p__Armatimonadetes;c__Armatimonadia;o__Armatimonadales	0,1	0,2	0,2	0,2	0,2	0,2	0	0	0	0,1	0	0	0,1	0	0	0,1
k__Bacteria;p__Armatimonadetes;c__Chthonomonadetes;o__Chthonomonadales	0,1	0,2	0,1	0,1	0,1	0,1	0	0	0	0	0	0	0	0	0,1	0,1
k__Bacteria;p__Armatimonadetes;c__[Fimbriimonadia];o__[Fimbriimonadales]	0,2	0,1	0,2	0,1	0,2	0,1	0,1	0,1	0,1	0,1	0,1	0,1	0,1	0,1	0,2	0,1
k__Bacteria;p__Bacteroidetes;Other;Other	0,3	0,2	0,3	0,3	0,3	0,4	0,4	0,1	0	0,1	0,3	0,1	0,1	0,1	0,1	0,4
k__Bacteria;p__Bacteroidetes;c__Bacteroidia;o__Bacteroidales	0,4	0,2	0,9	0,1	0,2	0,4	0,7	1	1,6	2,1	0,2	0,3	1,3	1,3	0,6	0,7
k__Bacteria;p__Bacteroidetes;c__Cytophagia;o__Cytophagales	1,7	1	1,8	1,3	3,4	2,3	0,1	0,4	0,4	1,7	0,7	0,8	0,8	0,4	0,5	2,6
k__Bacteria;p__Bacteroidetes;c__Flavobacteriia;o__Flavobacteriales	0,7	0,2	0,5	0,4	1,2	0,2	0,1	0,1	0	0,1	0	0,6	0,6	0,1	0,2	0,2
k__Bacteria;p__Bacteroidetes;c__Sphingobacteriia;o__	0,2	0,2	0,2	0,2	0,4	0,1	0,5	0,5	0	0,1	0	0,1	0,1	0	0,2	0,1

_Sphingobacteriales																
k__Bacteria;p__Bacteroidetes;c__[Rhodothermi];o__[Rhodothermales]	0	0,2	0,3	0,2	0	0,1	0,4	0,1	0,1	0,1	0	0,1	0,1	0	0	0,2
k__Bacteria;p__Bacteroidetes;c__[Saprospirae];o__[Saprospirales]	3,4	2,6	2,8	2,8	3,8	2,4	0,6	0,5	0,4	3,3	0,5	0,6	0,7	0,2	1,2	2,4
k__Bacteria;p__Chloroflexi;Other;Other	0,1	0,4	0,4	0,3	0,1	0,5	0,2	0,6	0,5	0,1	0,6	0,5	0,7	0,5	0,1	0,6
k__Bacteria;p__Chloroflexi;c__;o__	0	0,1	0,3	0,2	0	0,1	0	0,2	0,1	0	0	0,1	0,1	0,4	0,1	0,1
k__Bacteria;p__Chloroflexi;c__Anaerolineae;Other	0	0	0,1	0	0	0,1	2,6	0,1	0,1	0	0,4	0,1	0,1	0,1	0	0,1
k__Bacteria;p__Chloroflexi;c__Anaerolineae;o__	0	0,1	0,1	0,1	0	0,1	0	0	0	0,1	0	0	0	0,3	0	0
k__Bacteria;p__Chloroflexi;c__Anaerolineae;o__Ardenscatenales	0,1	0,6	0,7	0,6	0,1	0,7	0,4	0,9	0,2	0	0,1	0,5	0,7	0,9	0	0,8
k__Bacteria;p__Chloroflexi;c__Anaerolineae;o__CFB-26	0,1	0	0	0	0,1	0	0	0	0,1	0	0	0	0	0	0,3	0
k__Bacteria;p__Chloroflexi;c__Anaerolineae;o__Caldilineales	0,5	0,4	0,4	0,4	0,5	0,5	2,7	0,5	0,1	0,8	0,1	0,4	0,6	0,1	0,5	0,6
k__Bacteria;p__Chloroflexi;c__Anaerolineae;o__H39	0,1	0,1	0	0,1	0,1	0	0	0	0,2	0,1	0	0	0	0	0	0
k__Bacteria;p__Chloroflexi;c__Anaerolineae;o__S0208	0,2	0,1	0,1	0,1	0,2	0	0,1	0	0,1	0,2	0	0	0	0	0,2	0
k__Bacteria;p__Chloroflexi;c__Anaerolineae;o__SBR1031	0,7	1,2	1,1	1,1	1,3	3,4	16,1	1,2	2,2	0,8	8,5	1,3	2,1	6,3	0,6	2,2
k__Bacteria;p__Chloroflexi;c__Anaerolineae;o__SJA-15	0	0	0	0	0	0	0,2	0	0	0	0	0	0	0	0	0
k__Bacteria;p__Chloroflexi;c__C0119;o__	0,2	0,3	0,4	0,3	0,2	0,5	0,6	0,6	0	0	0,1	0,3	0,5	0,1	0,3	0,3
k__Bacteria;p__Chloroflexi;c__Chloroflexi;Other	0	0,1	0,1	0,1	0	0,8	0	1,3	0	0	0	0,5	0,5	0	0	1,2
k__Bacteria;p__Chloroflexi;c__Chloroflexi;o__AKIW781	0,5	0,8	0,9	0,9	0,6	1,6	0,1	0,8	0	0,2	0	0,7	0,9	0,1	0,1	1,3
k__Bacteria;p__Chloroflexi;c__Chloroflexi;o__Chloroflexales	0,2	1,4	1,5	1,5	0,5	1	0,4	0,9	0	0,5	0	0,8	1	0,4	0	1,3
k__Bacteria;p__Chloroflexi;c__Chloroflexi;o__[Roseiflexales]	0,5	0,2	0,2	0,3	0,7	0,1	0	0,1	0,2	0,6	0	0,1	0,1	0	0,2	0,2
k__Bacteria;p__Chloroflexi;c__Ellin6529;o__	1,3	0,8	0,9	0,9	1,1	0,5	0,1	0,4	0,8	2,8	0,2	0,7	0,9	0,3	1,2	0,6
k__Bacteria;p__Chloroflexi;c__Gitt-GS-136;o__	0,1	0,2	0,2	0,2	0	0,1	0	0,1	0	0,2	0	0,2	0,2	0,1	0,1	0,2
k__Bacteria;p__Chloroflexi;c__Ktedonobacteria;Other	0	0,2	0	0	0	0,7	0,7	0,8	26,4	0,1	22,1	0,2	0,2	0,9	0	0,9
k__Bacteria;p__Chloroflexi;c__Ktedonobacteria;o__Thermogemmatisporales	0	0,2	0	0	0	0,8	2	1,9	13,3	0,1	24,1	1,2	1,4	0,4	0	5,6
k__Bacteria;p__Chloroflexi;c__S085;o__	0,2	0,2	0,2	0,1	0,2	0,1	0	0,1	0,1	0,2	0	0,1	0,1	0	0,3	0,1
k__Bacteria;p__Chloroflexi;c__TK10;o__AKYG885	0,3	0,3	0,3	0,3	0,2	0,2	0,1	0,1	0,2	0,1	0	0	0,1	0	0,2	0,2
k__Bacteria;p__Chloroflexi;c__TK10;o__B07_WMSP1	0,2	0,1	0,1	0,1	0,1	0,2	0	0	0	0	0,4	0,1	0,2	0,8	0,3	0,2
k__Bacteria;p__Chloroflexi;c__TK17;o__mle1-48	0,1	0,1	0,1	0,1	0,1	0	0	0	0,1	0,2	0	0	0	0	0,1	0
k__Bacteria;p__Chloroflexi;c__Thermomicrobia;o__AKYG1722	0,1	0,1	0,1	0,1	0,2	0,1	0	0,1	0	0,4	0	0,2	0,2	0	0,1	0,1
k__Bacteria;p__Chloroflexi;c__Thermomicrobia;o__JG30-KF-CM45	1	1,2	1,7	1,7	0,7	1,8	1,9	2	1	2,2	1	1,7	1,6	2,7	0,8	2
k__Bacteria;p__Cyanobacteria;Other;Other	0	0,1	0,1	0,2	0,1	0,5	0	0,4	0	0	0	0,2	0,4	0,1	0	0,4
k__Bacteria;p__Cyanobacteria;c__Chloroplast;o__Stramenopiles	0,2	0	0	0	0,1	0,1	0	0	0	0	0	0	0	0	0,2	0
k__Bacteria;p__Cyanobacteria;c__Chloroplast;o__Streptophyta	0,3	0	0	0	0	0	0	0	0	0	0	0	0	0	0	0,1
k__Bacteria;p__Cyanobacteria;c__Nostocophycideae;o__Nostocales	0	0	0,1	0,3	0,2	0,1	0	0,1	0	0	0	0	0,1	0	0	0,1
k__Bacteria;p__Cyanobacteria;c__Oscillatoriophycideae;o__Chroococcales	0	0,2	0,1	0,2	0,1	0,1	0	0,8	0	0	0	0,1	0,2	0,1	0	0,1
k__Bacteria;p__Cyanobacteria;c__Oscillatoriophycideae;o__Oscillatoriales	0,2	0	0	0	0,1	0,2	0	0,2	0	0	0	0	0	0,1	0	0,4
k__Bacteria;p__Cyanobacteria;c__Synechococcophycideae;Other	0	0	0	0	0	0,1	0	0,5	0	0	0	0	0,2	0,2	0	0,2
k__Bacteria;p__Cyanobacteria;c__Synechococcophycideae;o__Pseudanabaenales	1,1	0,2	0,4	0,5	0,5	1,2	0	1,1	0	0,1	0	0,1	0,3	0,3	0	1,5
k__Bacteria;p__Firmicutes;c__Bacilli;o__Bacillales	1,1	0,6	0,5	0,4	0,7	0,4	2,3	0,6	0,3	2,6	0,1	0,8	1,1	0,5	4,6	0,3
k__Bacteria;p__Firmicutes;c__Clostridia;Other	0	0	0	0	0	0	0	0,5	0	0	0	0,1	0,3	0	0	0

k__Bacteria;p__Firmicutes;c__Clostridia;o__Clostridiales	0,4	0,3	0,3	0,1	0,1	0,3	3,1	0,7	1,3	1,8	0,2	0,3	0,7	0,6	1,3	0,3
k__Bacteria;p__Firmicutes;c__Clostridia;o__SHA-98	0,3	0	0	0	0	0	0	0	0	0	0	0	0	0	0	0
k__Bacteria;p__Firmicutes;c__Erysipelotrichi;o__Erysipelotrichales	0	0	0	0	0	0	0	0,4	0	0	0	0	0	0	0	0
k__Bacteria;p__Gemmatimonadetes;c__Gemm-1;o__	1,1	0,5	0,3	0,4	0,7	0,3	0	0	0	0,4	0	0,1	0,1	0	1	0,2
k__Bacteria;p__Gemmatimonadetes;c__Gemm-2;o__	0	0	0	0	0	0	0	0	0	0,1	0	0	0	0	0,1	0
k__Bacteria;p__Gemmatimonadetes;c__Gemm-3;o__	0,3	0,3	0,3	0,3	0,4	0,2	0,1	0	0	0,7	0	0,1	0,1	0	0,1	0,2
k__Bacteria;p__Gemmatimonadetes;c__Gemm-5;o__	0,3	0,2	0,1	0,1	0,1	0,1	0	0	0,1	0,1	0,2	0,1	0,1	0	0,5	0,1
k__Bacteria;p__Gemmatimonadetes;c__Gemmatimonadetes;Other	0,3	0,2	0,3	0,3	0,3	0,3	0	0	0,2	0,3	0,1	0,1	0,1	0	0	0,3
k__Bacteria;p__Gemmatimonadetes;c__Gemmatimonadetes;o__	0,4	0,4	0,3	0,2	0,3	0,5	0,3	0,1	0,1	0,5	2	0,2	0,2	0,5	0,4	0,4
k__Bacteria;p__Gemmatimonadetes;c__Gemmatimonadetes;o__C114	0,1	0	0	0	0	0	0	0	0	0	0	0	0	0	0,2	0
k__Bacteria;p__Gemmatimonadetes;c__Gemmatimonadetes;o__Gemmatimonadales	0,3	0,4	0,4	0,3	0,4	0,5	0	0,1	0,1	0,5	0	0,2	0,2	0	0,1	0,4
k__Bacteria;p__Gemmatimonadetes;c__Gemmatimonadetes;o__N1423WL	0,2	0	0	0	0,1	0	0	0	0	0,1	0	0	0	0	0,1	0
k__Bacteria;p__Nitrospirae;c__Nitrospira;o__Nitrospirales	0,5	0,2	0,1	0,1	0,4	0,1	0	0,1	0	0,3	0	0,1	0	0	2,2	0,1
k__Bacteria;p__OD1;c__SM2F11;o__	0	0	0	0	0	0	0	0,3	0	0	0	0	0	0	0	0
k__Bacteria;p__OD1;c__ZB2;o__	0	0	0	0	0	0	0	0,6	0	0	0,1	0	0	0	0	0,1
k__Bacteria;p__Planctomycetes;c__OM190;o__agg27	0,1	0,1	0,1	0,2	0,1	0	0	0	0	0,1	0	0	0	0	0,2	0
k__Bacteria;p__Planctomycetes;c__Phycisphaerae;o__Phycisphaerales	0,1	0,2	0,2	0,2	0,1	0,1	2,7	0,1	0,2	0,1	0,7	0,1	0,1	0,1	0,2	0,1
k__Bacteria;p__Planctomycetes;c__Phycisphaerae;o__WD2101	1,4	1,2	1,1	1,1	1,2	0,8	1,2	0,4	0,8	1,5	0,2	0,5	0,4	0,4	0,5	0,9
k__Bacteria;p__Planctomycetes;c__Phycisphaerae;o__mle1-8	0	0	0	0	0	0	0	0	0	0	0	0	0	0,2	0	0
k__Bacteria;p__Planctomycetes;c__Planctomycetia;o__Gemmatales	1,5	2	3,1	2,6	1,5	1,7	1,7	0,8	2,1	2,7	1,6	0,8	1,1	2,5	1,9	1,8
k__Bacteria;p__Planctomycetes;c__Planctomycetia;o__Pirellulales	1,8	1,6	1,6	1,4	1,7	1,1	0,3	0,4	0,3	1,7	0,2	0,6	0,7	0,2	2	1,1
k__Bacteria;p__Planctomycetes;c__Planctomycetia;o__Planctomycetales	0,5	0,5	0,6	0,5	0,5	0,5	0,1	0,1	0,1	0,5	0	0,1	0,2	0,1	0,3	0,6
k__Bacteria;p__Proteobacteria;Other;Other	0,2	0,3	0,3	0,2	0,2	0,3	0,1	0,5	0,3	0,1	0,1	0,3	0,2	0,5	0,1	0,3
k__Bacteria;p__Proteobacteria;c__Alphaproteobacteria;Other	0,2	0,4	0,5	0,6	0,2	0,5	0,7	0,9	0,7	0,4	0,5	0,8	0,7	0,8	0,2	0,5
k__Bacteria;p__Proteobacteria;c__Alphaproteobacteria;o__	0,1	0,1	0	0,1	0,2	0	0	0,1	0,2	0	0,1	0	0	0	0,1	0
k__Bacteria;p__Proteobacteria;c__Alphaproteobacteria;o__BD7-3	0	0,1	0,1	0,1	0,1	0,2	0,1	0,4	0	0	0	0,1	0,1	0	0	0,3
k__Bacteria;p__Proteobacteria;c__Alphaproteobacteria;o__Caulobacterales	0,5	0,8	1,2	1,2	0,6	1,9	1,7	1,2	0,1	0,6	0,6	2,9	1,6	1	0,1	2,2
k__Bacteria;p__Proteobacteria;c__Alphaproteobacteria;o__Rhizobiales	7,8	8,5	8,3	7,6	9,2	7	11,9	3,9	7,8	8,4	3,4	5,1	7,6	2,8	3,9	6,5
k__Bacteria;p__Proteobacteria;c__Alphaproteobacteria;o__Rhodobacterales	1,4	1,8	2,1	2,3	1,7	2,3	5,2	6,3	3,5	2,3	0,6	7,4	5,8	2,8	0,6	2,3
k__Bacteria;p__Proteobacteria;c__Alphaproteobacteria;o__Rhodospirillales	1,4	2,1	2,3	2,4	1,6	1,6	4,4	1,4	2,8	3,7	0,8	1,7	1,9	2	0,8	1,6
k__Bacteria;p__Proteobacteria;c__Alphaproteobacteria;o__Rickettsiales	0,1	0,1	0,1	0,1	0,4	0,1	0,1	0,4	0	0	0	0	0	0	0	0,1
k__Bacteria;p__Proteobacteria;c__Alphaproteobacteria;o__Sphingomonadales	3	3	3,6	3,8	2,5	4	1,2	1,2	0,6	2,3	0,6	8,1	5,3	2,1	0,6	3,8
k__Bacteria;p__Proteobacteria;c__Betaproteobacteria;Other	0,6	0,5	0,4	0,3	0,4	0,4	0	0,2	0	0,8	0,1	0,5	0,3	0,5	0,3	0,4
k__Bacteria;p__Proteobacteria;c__Betaproteobacteria;o__	1	0,6	0,4	0,5	0,9	0,5	0	0	0	0,2	0	0,1	0,1	0	0,8	0,3
k__Bacteria;p__Proteobacteria;c__Betaproteobacteria	3,7	4,1	5,1	3,6	4,8	8,2	1	9,5	1,5	4,1	0,5	23,5	13,7	12,1	1,3	8,1

;o__Burkholderiales																
k__Bacteria;p__Proteobacteria;c__Betaproteobacteria;o__Ellin6067	1,2	0,4	0,2	0,3	0,7	0,4	0	0	0,2	0,4	0	0,1	0,2	0	0,5	0,3
k__Bacteria;p__Proteobacteria;c__Betaproteobacteria;o__MND1	0,7	0,3	0,1	0,1	0,5	0,2	0	0	0,1	0,5	0	0	0	0	1,2	0,2
k__Bacteria;p__Proteobacteria;c__Betaproteobacteria;o__Methylophilales	0	0	0	0	0	0,1	0	0,3	0	0	0	1,4	0,8	0,4	0	0,1
k__Bacteria;p__Proteobacteria;c__Betaproteobacteria;o__Rhodocyclales	0,3	0,4	0,1	0,1	0,2	0,1	0	0,3	0	0,2	0	1	0,4	0,1	0,1	0,1
k__Bacteria;p__Proteobacteria;c__Betaproteobacteria;o__SC-I-84	0,3	0,3	0,3	0,3	0,2	0,1	0	0	0,1	0,6	0	0,1	0,1	0	0,1	0,1
k__Bacteria;p__Proteobacteria;c__Deltaproteobacteria;o__Bdellovibrionales	0,1	0,1	0,1	0,1	0,2	0,2	0,1	0,2	0	0,1	0,1	0,2	0,2	0,1	0,1	0,2
k__Bacteria;p__Proteobacteria;c__Deltaproteobacteria;o__Desulfobacterales	0	0	0	0	0	0	0	0	0	0,3	0	0	0	0	0	0
k__Bacteria;p__Proteobacteria;c__Deltaproteobacteria;o__Desulfuromonadales	0,1	0,1	0	0	0	0	0	0	0,1	0,2	0	0	0	0	0	0
k__Bacteria;p__Proteobacteria;c__Deltaproteobacteria;o__MIZ46	0,2	0,1	0,1	0,1	0,2	0,1	0	0	0	0,1	0	0	0	0	0,2	0,1
k__Bacteria;p__Proteobacteria;c__Deltaproteobacteria;o__Myxococcales	2	1,7	1,7	1,7	2,3	1,1	0,8	0,5	2,7	2,6	3,1	0,7	0,9	5,3	2,1	1,2
k__Bacteria;p__Proteobacteria;c__Deltaproteobacteria;o__Syntrophobacterales	0,4	0,1	0,1	0,1	0,4	0,1	0,1	0	0,2	0,5	0,1	0	0,1	0,1	1,4	0
k__Bacteria;p__Proteobacteria;c__Deltaproteobacteria;o__[Entotheonellales]	0	0	0	0	0	0	0	0	0	0	0	0	0	0	0,9	0
k__Bacteria;p__Proteobacteria;c__Gammaproteobacteria;Other	4,6	5,7	2,7	2,1	1,9	2,7	0,2	1	0,6	0,3	0,2	1,1	1,4	0,4	3	1,8
k__Bacteria;p__Proteobacteria;c__Gammaproteobacteria;o__Alteromonadales	0,2	0,1	0,1	0,1	0,3	0	0	1,8	0	0	0	0,3	0,2	0,1	0	0
k__Bacteria;p__Proteobacteria;c__Gammaproteobacteria;o__Legionellales	0,1	0,1	0,1	0,2	0,2	0,1	0,1	0,4	0	0,1	0	0,3	0,1	0,1	0,1	0,1
k__Bacteria;p__Proteobacteria;c__Gammaproteobacteria;o__Methylococcales	3,4	7,1	3,3	2,9	2,4	1,6	7,6	0,8	1,8	0,1	0,9	0,9	4,3	0,4	0,6	1
k__Bacteria;p__Proteobacteria;c__Gammaproteobacteria;o__Pseudomonadales	0	0	0,1	0,1	0	0,1	0,1	0,3	0	0	0	0,5	0,3	0,1	0	0
k__Bacteria;p__Proteobacteria;c__Gammaproteobacteria;o__Thiotrichales	0,1	0,1	0,1	0,1	0,2	0	0	0	0	0,2	0	0	0	0	0,1	0
k__Bacteria;p__Proteobacteria;c__Gammaproteobacteria;o__Xanthomonadales	0,9	0,6	1,4	1,5	1,7	1	0,5	14,6	1,9	1,2	0,1	8,4	5,3	4,1	1,3	1,5
k__Bacteria;p__TM7;c__SC3;o__	0,1	0,1	0,1	0	0	0,1	0	0	0	0	0,2	0,1	0,1	0,3	0	0,1
k__Bacteria;p__TM7;c__TM7-1;o__	0,6	0,7	0,6	0,5	0,5	0,8	0,4	1,1	0,1	0,4	0,7	0,6	0,9	0,5	0,2	0,7
k__Bacteria;p__TM7;c__TM7-3;o__EW055	0	0	0	0	0	0,1	0,1	0,2	0	0	0	0	0	0	0	0
k__Bacteria;p__Verrucomicrobia;c__Opitutae;o__Opitutales	0,2	0,1	0,1	0,1	0,3	0,1	0,1	0,1	0	0,2	0	0	0	0	0,1	0,1
k__Bacteria;p__Verrucomicrobia;c__Verrucomicrobiae;o__Verrucomicrobiales	0,2	0,1	0,4	0,4	0,3	0,6	0	0,1	0	0,2	0	0,2	0,1	0,1	0,1	0,8
k__Bacteria;p__Verrucomicrobia;c__[Methylacidiphilae];o__Methylacidiphilales	0	0	0	0,1	0	0	0	0	0,2	0	1,6	0	0	0	0	0,3
k__Bacteria;p__Verrucomicrobia;c__[Pedosphaerae];o__[Pedosphaerales]	0,6	0,3	0,2	0,2	0,6	0,2	0	0	0,2	0,5	0	0	0,1	0	0,6	0,1
k__Bacteria;p__Verrucomicrobia;c__[Spartobacteria];o__[Chthoniobacterales]	1,6	1,9	2,5	2,4	1,3	1,7	0,3	0,5	1,4	0,8	1	0,4	0,5	0,9	0,5	1,5
k__Bacteria;p__WS3;c__PRR-12;o__Sediment-1	0,1	0	0	0	0,1	0	0	0	0	0,1	0	0	0	0	0,7	0
k__Bacteria;p__[Thermi];c__Deinococci;o__Deinococcales	0	0,1	0,1	0,2	0,1	0,8	0,1	1,1	0	0	0	0,6	2,4	0,2	0	0,8
k__Bacteria;p__[Thermi];c__Deinococci;o__Thermales	0	0	0	0	0	0	0	0,3	0	0	0	0	0,7	0	0	0

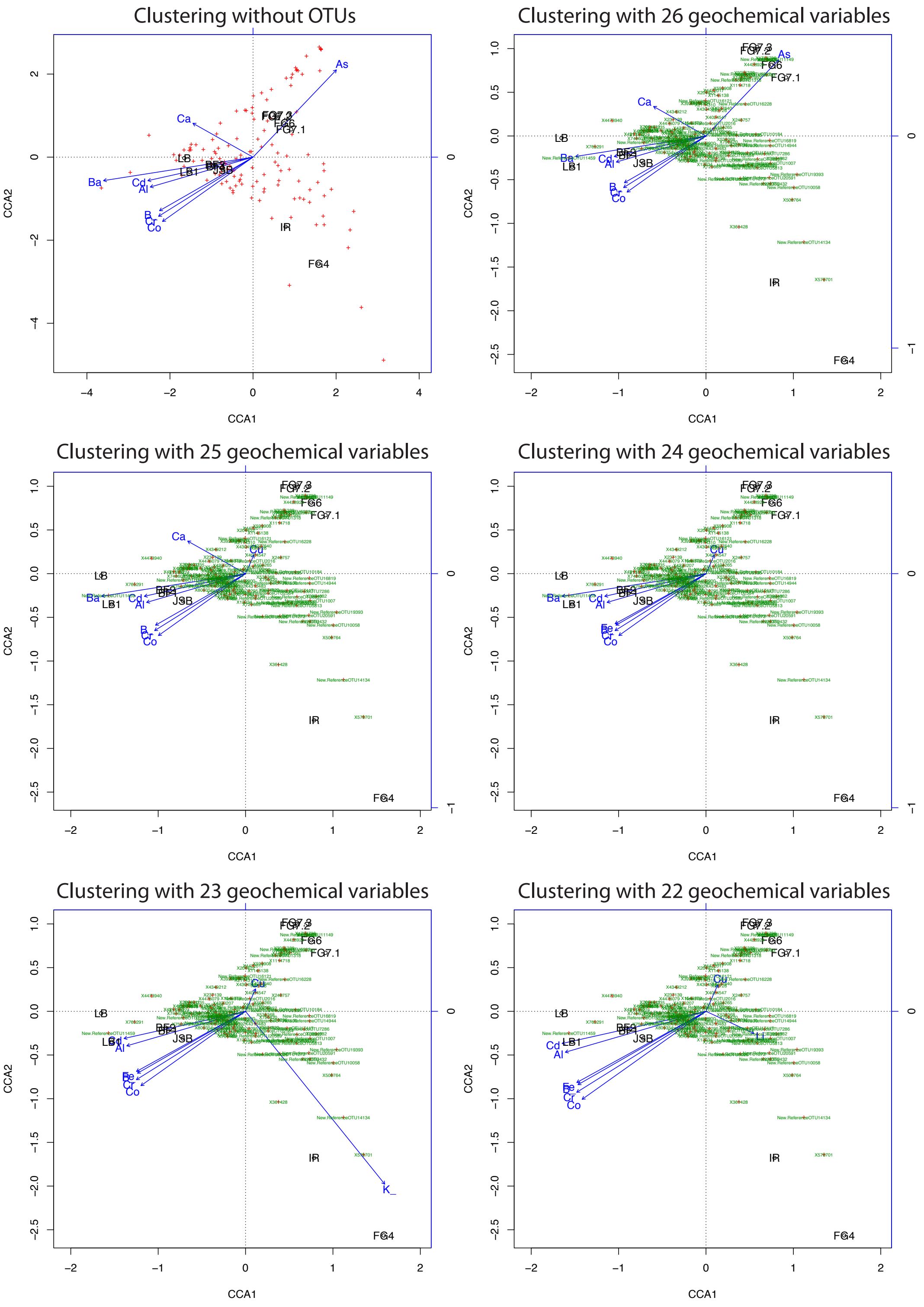

Fig. S5. CCA plot showing correlation between OTUs and geochemical metadata using 26-22 variables.

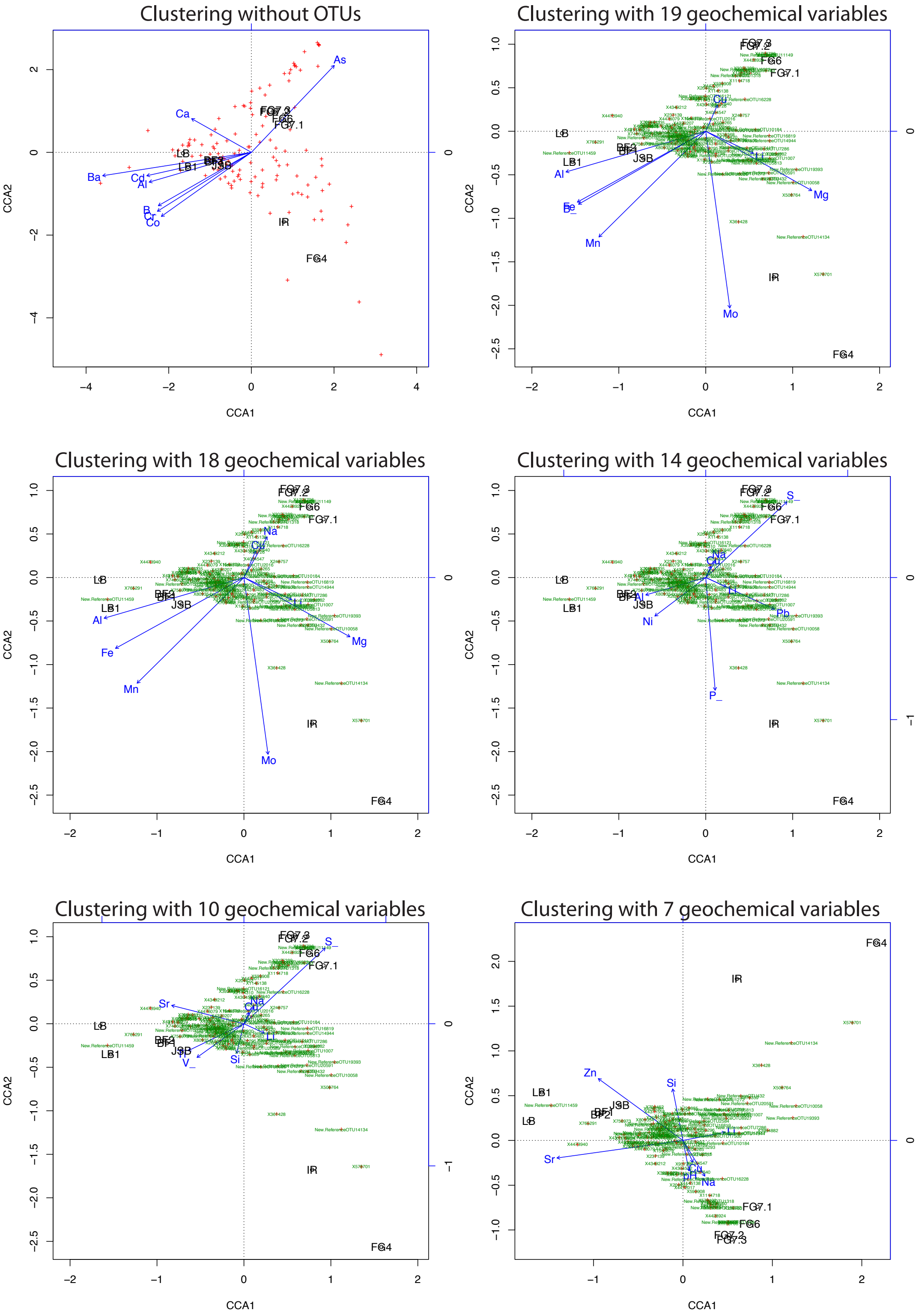

Fig. S6. CCA plot showing correlation between OTUs and geochemical metadata using 19-7 variables.

References

Robertson, A. H. F., and N. H. Woodcock. "Sedimentation in Oblique-Slip Mobile Zones." (1980): pp.127-145.

Hosgörmez, H., Etiope, G. & Yalcin, M.N., 2008. New evidence for a mixed inorganic and organic origin of the Olympic Chimaera fire (Turkey): a large onshore seepage of abiogenic gas. Geofluids, 8(4), pp.263–273.
